# Integrated transcriptomic and Mendelian randomization analysis identifies novel biomarkers for type 2 diabetes–associated cardiac dysfunction: cynaropicrin as a candidate intervention

**DOI:** 10.1007/s00210-026-05585-2

**Published:** 2026-07-10

**Authors:** Lindong Zhang, Lingling Qin, Lili Wu, Tonghua Liu

**Affiliations:** 1 School of Traditional Chinese Medicine, Beijing University of Chinese Medicine, Beijing, 102488 China; 2 Key Laboratory of Health Cultivation of the Ministry of Education, Beijing University of Chinese Medicine, Beijing, 102488 China; 3 Key Laboratory of Health Cultivation of Beijing, Beijing University of Chinese Medicine, Beijing, 102488 China; 4 Science and Technology Office, Beijing University of Chinese Medicine, Beijing, 102488 China

**Keywords:** Type 2 diabetes mellitus, Diabetic cardiac dysfunction, Biomarkers, Cynaropicrin, Mendelian randomization

## Abstract

The objective of this study is to identify T2DM-associated biomarkers linked to diabetic cardiac dysfunction-related transcriptomic signatures through integrated transcriptomic and Mendelian randomization analysis and evaluate cynaropicrin as a candidate cardiometabolic intervention. Transcriptomic datasets from the GEO database related to diabetic cardiac dysfunction, heart failure, and cardiomyopathy-associated phenotypes were processed for differential expression analysis and WGCNA, with consideration of disease-definition and tissue-source heterogeneity. A two-sample Mendelian randomization analysis was used to evaluate genetically predicted associations between candidate gene expression and T2DM risk. External validation assessed diagnostic performance via ROC analysis. Multi-database screening and molecular docking identified candidate compounds. Streptozotocin-induced diabetic rats were treated with cynaropicrin for 8 weeks, with comprehensive cardiac and metabolic assessment. Analysis identified 30 candidate genes with functional enrichment in proteostasis and metabolism. Mendelian randomization prioritized three T2DM-associated genetically supported genes: PPIP5K2, RBM23, and IGF2BP2. Cynaropicrin significantly improved cardiac function, reduced fibrosis, and enhanced glycemic control in diabetic rats while suppressing pro-fibrotic signaling. PPIP5K2, RBM23, and IGF2BP2 represent T2DM-associated genetically supported biomarkers dysregulated in diabetic cardiac dysfunction-related datasets with diagnostic potential. Cynaropicrin demonstrates candidate cardioprotective and metabolic benefits in diabetic rats; however, its direct molecular targets and mechanism of action require further validation.

**Supplementary Information:**

The online version contains supplementary material available at https://doi.org/10.1007/s00210-026-05585-2.

## Introduction

Type 2 diabetes mellitus (T2DM) has become a major global health challenge, with cardiovascular complications representing the leading cause of mortality in affected patients (Adamo et al. 2020). Diabetic cardiomyopathy (DCM), a serious complication of T2DM, is characterized by pathological changes including myocardial fibrosis, ventricular remodeling, and functional dysfunction (Aragam and Natarajan 2020). Recent studies have demonstrated that T2DM patients exhibit significantly increased risk of heart failure, with this association showing independent causality through Mendelian randomization analyses (Ben Salem et al. 2017). Therefore, early identification of molecular mechanisms underlying T2DM-related cardiac dysfunction and remodeling phenotypes and discovery of candidate therapeutic strategies are crucial for improving patient outcomes.

With the advancement of high-throughput sequencing technologies, transcriptomic analysis has emerged as a powerful tool for elucidating disease molecular mechanisms. Recent studies have successfully identified key molecular biomarkers associated with DCM or diabetic cardiac dysfunction-related phenotypes by integrating multiple gene expression datasets using weighted gene co-expression network analysis (WGCNA) and differential expression analysis (Bowden et al. 2015; Conning-Rowland et al. 2024; Cui et al. 2024a). Liu et al. (Cui et al. 2024a) integrated two GEO datasets (GSE106180 and GSE161827), combining differential gene expression analysis, WGCNA, and immune infiltration analysis to construct a diagnostic model for DCM. Similarly, a study published in*Cardiovascular Research*(Cui et al. 2024b) identified Erbb3 and Hspa2 as potential biomarker pairs for cardiomyopathy risk prediction through diabetic myocardial transcriptome analysis, validated using the UK Biobank cardiac magnetic resonance data for heart failure outcomes. These studies have established important paradigms for the “transcriptome-phenotype-clinical” research framework.

However, traditional association studies cannot establish causality. Mendelian randomization (MR), which utilizes genetic variants as instrumental variables for causal inference, effectively avoids confounding factors and reverse causation (Ebrahimi-Mameghani et al. 2018). In the present study, MR was applied to assess whether genetically proxied expression of candidate genes was associated with T2DM risk, thereby supporting the genetic prioritization of T2DM-associated candidate genes. Larsson et al. (Elsebai et al. 2016) demonstrated through two-sample MR analysis that T2DM genetic susceptibility causally increases heart failure risk, providing key evidence for the “diabetes-heart failure causal chain.” Additionally, a recent study in*Communications Medicine*(Emdin et al. 2017) utilized 21 cardiac MRI parameters as MR instrumental variables, directly supporting the “remodeling phenotype → outcome” causal bridge. A recent*Cell Reports Medicine*study (Frangogiannis 2021) employed transcriptome-wide Mendelian randomization (TWMR) to identify potential drug targets for heart failure, providing direct reference for the “eQTL → disease” gene prioritization framework.

Regarding candidate genes, RNA-binding proteins and splicing regulators play crucial roles in cardiac remodeling. IGF2BP2 is upregulated in the heart under stress conditions and has been shown to contribute to the development of dilated cardiomyopathy (Gao and Dudley 2013). RBM24 functions as a key splicing regulator affecting myofibril assembly and cardiac structure (Grarup et al. 2007). These findings provide strong evidence for the role of RNA-binding protein genes in cardiac remodeling.

In terms of therapeutic strategies, myocardial fibrosis represents the core pathological process of DCM, with TGF-β signaling pathway inhibition considered a central anti-fibrotic strategy (Huang et al. 2023; Jia et al. 2018). Natural products show potential in cardiovascular disease treatment due to their multi-target biological activities. Cynaropicrin, a natural sesquiterpene lactone compound derived from artichoke, exhibits significant anti-inflammatory, antioxidant, and anti-fibrotic activities (Krumbein et al. 2023). Studies have demonstrated that cynaropicrin exerts anti-inflammatory effects through NF-κB signaling pathway inhibition (Langfelder and Horvath 2008)and can activate the Nrf2 pathway to alleviate oxidative stress (Larsson et al. 2020; Liu et al. 2025). Clinical data indicate that artichoke leaf extract exerts measurable metabolic benefits, such as reducing circulating oxidized LDL in patients with metabolic syndrome (Lopaschuk et al. 2021), supporting its potential relevance to cardiometabolic regulation.

Although previous studies have explored DCM molecular mechanisms and natural product therapeutic potential, comprehensive studies integrating transcriptomics, Mendelian randomization, and experimental validation remain rare. Furthermore, the specific mechanisms of cynaropicrin in T2DM-associated cardiac dysfunction have not been systematically investigated. Therefore, this study aims to: (Adamo et al. 2020)identify key biomarkers associated with diabetic cardiac dysfunction-related transcriptomic signatures and establish predictive models through integrated transcriptomic and Mendelian randomization analyses; (Aragam and Natarajan 2020)discover cynaropicrin as a candidate compound through multi-database screening and molecular docking; (Ben Salem et al. 2017) validate key gene expression changes and cynaropicrin cardiometabolic effects in T2DM rat models. This study is expected to provide theoretical and experimental support for improving early diagnosis and candidate therapeutic strategies for DCM.

## Materials and methods

### Bioinformatic analysis

Publicly available transcriptomes related to diabetic cardiac dysfunction and cardiomyopathy-associated phenotypes were retrieved from GEO (e.g., GSE288708, GSE197850). Raw counts (RNA-seq) or probe-level intensities (microarray) and sample annotations were imported into R (v4.x). Platform probes were mapped to official HGNC symbols using the corresponding GPL files; duplicated symbols were summarized by the median. For RNA-seq, counts were filtered (CPM > 1 in ≥ 50% of samples), TMM-normalized (edgeR) and transformed with voom (limma). Microarray intensities were log2-transformed and quantile-normalized when appropriate. When integrating multiple datasets, batch effects were assessed by PCA/UMAP and corrected with ComBat (sva) while preserving group labels. Obvious outliers (± 4 SD on the first two PCs or sample network connectivity *Z*<  − 2) were excluded prior to modeling. Differential expression between disease and control was assessed with limma. A design matrix encoding group status was fitted gene-wise; empirical Bayes variance moderation (eBayes) was applied. Multiple testing was controlled by Benjamini–Hochberg FDR. Functional annotation of DEGs was performed using clusterProfiler for Gene Ontology (GO) analyses, including biological process, cellular component, and molecular function, as well as KEGG pathway analysis. The background consisted of all genes tested for differential expression. To estimate pathway-level fluxes and metabolite activities, we used METAFlux (human genome-scale metabolic model; default parameters (Lu et al. 2022). Disease vs control expression matrices were supplied to infer reaction activity scores and metabolite production/consumption tendencies. A signed co-expression network was built with WGCNA on the variance-stabilized expression matrix (top 5000–10,000 most variable genes). The soft-thresholding power (β) was chosen where the scale-free topology fit (signed*R*^2^) reached a plateau (typically *R*^2^ ≥ 0.8) while maintaining adequate mean connectivity. Adjacency matrices were transformed to topological overlap (TOM) and genes were hierarchically clustered. Modules were detected by dynamic tree cut (minModuleSize = 30–50) and merged at eigengene correlation > 0.75. Module–trait associations were quantified by Pearson correlations between module eigengenes and case–control status. Disease-associated modules (|*r*|≥ 0.4 with *P* < 0.05) were intersected with the DEG catalog to obtain a candidate set for genetic prioritization and translational analyses.

### Mendelian randomization

To prioritize T2DM-associated genetically supported genes, we instrumented cis-gene expression (± 500 kb around each TSS) using cis-eQTL summary statistics (e.g., GTEx v8 left ventricle/atrial appendage). Independent instruments were selected at *P* < 5 × 10^−^⁸, LD *r*^2^ < 0.01 within 10-Mb window (1000 Genomes European reference), removing palindromic ambiguous SNPs. Two-sample MR was performed against a large BMI-adjusted T2D GWAS (summary-level data) using TwoSampleMR. Primary inference used inverse-variance weighted (IVW); robustness was assessed with MR-Egger, weighted median, and weighted mode. Sensitivity analyses included Cochran’s Q (heterogeneity), the MR-Egger intercept (horizontal pleiotropy), MR-PRESSO (global/outlier tests), and Steiger directionality. Leave-one-out analyses assessed leverage by single instruments. Where regional LD and data availability permitted, we performed colocalization (coloc/SuSiE) to evaluate shared causal variants. Bonferroni or BH FDR was applied across tested genes. The MR outcome was BMI-adjusted T2DM risk; therefore, MR findings were interpreted as genetic evidence supporting associations between gene expression and T2DM susceptibility.

### External validation and diagnostic performance

Key genes from MR were evaluated in independent GEO cohorts (e.g., GSE25724) to determine whether T2DM-associated genetically supported genes were consistently dysregulated in diabetic cardiac dysfunction-related transcriptomic settings. Expression differences were tested by Student’s t or Wilcoxon tests as appropriate. Diagnostic performance was quantified by ROC curves using pROC; AUCs were compared by DeLong’s test and 95% CIs obtained by bootstrap resampling (2,000 iterations). Where relevant, multi-marker models (logistic regression with clinical covariates) were built and evaluated by calibration curves and decision-curve analysis.

### Immune infiltration (CIBERSORT)

Bulk expression matrices were deconvolved into 22 leukocyte subsets using CIBERSORT with the LM22 signature (1,000 permutations). For RNA-seq, quantile normalization was disabled; for arrays, it was left at default. Only samples with P (CIBERSORT) < 0.05 were retained for downstream comparison. Group differences in cell-type fractions were assessed using Wilcoxon tests with BH FDR control. Global relationships among subsets were assessed by Spearman’s correlation matrices.

### Single-gene GSEA

For each key gene (PPIP5K2, RBM23, IGF2BP2), samples were dichotomized at the median (high vs low), or all genes were ranked by their Spearman correlation with the key gene across samples. GSEA (clusterProfiler/fgsea) was run against MSigDB Hallmark and KEGG gene sets, using 10,000 permutations. Enriched pathways were reported at FDR *q* < 0.25 (GSEA default) and adjusted *P* < 0.05.

### Natural-product nomination and molecular docking

Drug/compound candidates were queried from CTDbase, DSigDB, and Coremine Medical, using (i) the disease DEG signature and (ii) the key-gene list. This strategy was intended for hypothesis generation and compound prioritization. Compounds consistently enriched across resources and annotated as anti-inflammatory/anti-fibrotic were shortlisted. Three-dimensional protein structures (PDB or AlphaFold2 models) of PPIP5K2, RBM23, and IGF2BP2 were prepared in AutoDockTools (polar hydrogens added; non-polar merged; Gasteiger charges). Cynaropicrin 3D coordinates were downloaded from PubChem and energy-minimized. Docking used AutoDock Vina (exhaustiveness 8–16) with grid boxes centered on predicted pockets (FPocket/P2Rank) or canonical clefts; the top-scoring poses were visualized in PyMOL. Binding affinity (kcal/mol) heatmaps were used to summarize results across targets.

### Animals and diabetic cardiomyopathy model

Male Sprague–Dawley (SD) rats, 6 weeks old, were obtained from an accredited vendor and housed under SPF conditions (12-h light/dark, 22–24 °C, ad libitum chow/water). Animals were randomly assigned to diabetic and non-diabetic streams. The diabetic stream received a high-fat diet (HFD) for 4 weeks, followed by streptozotocin (STZ) 35 mg kg^−1^ i.p. in 0.1 mol L^−1^ citrate buffer (pH 4.5). One week later, tail-vein fasting blood glucose (FBG) was measured; FBG ≥ 11.1 mmol L^−1^ qualified as type 2 diabetes mellitus (TADM). Non-diabetic rats were fed regular chow. All procedures were approved by the Institutional Animal Care and Use Committee (Approval No. D202505-16) and complied with the ARRIVE guidelines. Rats were randomized (block randomization; investigator-blinded outcome assessment) to four groups for 8 weeks: G1 (Control): regular diet + vehicle (physiological saline, gavage, once daily). G2 (Cyn): regular diet + cynaropicrin 20 mg kg^−1^ day^−1^ (gavage). G3 (STZ): HFD + STZ + vehicle (gavage). G4 (STZ + Cyn): HFD + STZ + cynaropicrin 20 mg kg^−1^ day^−1^ (gavage). The vehicle was matched in volume across groups. Body weight and FBG were monitored weekly.

### Tissue collection

At the endpoint, rats were anesthetized (isoflurane 2%) and euthanized. Hearts were excised, rinsed in ice-cold PBS, blotted dry, and weighed; tibia length (TL) was measured to compute HW/TL (mg mm^−1^). Left-ventricular (LV) tissue was snap-frozen in liquid nitrogen (molecular assays) or fixed in 4% paraformaldehyde (histology).

### Echocardiography

Cardiac function was assessed on a small-animal ultrasound system (e.g., Vevo 2100) under light isoflurane (1–1.5%). M-mode images at the papillary muscle level were acquired; EF, FS, LVEDD, and LVESD were computed from three consecutive cardiac cycles and averaged by a blinded operator.

### Serum biochemistry

Blood was collected by cardiac puncture after overnight fasting. Serum LDH, cTnT, CK-MB, ALT, AST, BUN, and Scr were quantified using commercial kits on an automated analyzer according to the manufacturers’ instructions.

### Metabolic ELISAs

FBG was measured by glucose oxidase method; fasting serum insulin (FSI) was measured by ELISA. HOMA-IR was calculated as (FBG × FSI)/22.5, using units of mmol L^−1^ and µU mL^−1^, respectively.

### qRT-PCR

Total RNA from LV tissue was extracted with TRIzol and DNase-treated. cDNA synthesis used random hexamers (mRNA). qPCR was performed with SYBR Green on an ABI 7500. Primer sequences for PPIP5K2 and IGF2BP2 are listed in Table S1. Reactions were run in technical triplicate; GAPDH served as reference. Relative expression was computed by 2^ − ΔΔCt.

### Western blotting

LV tissue was homogenized in RIPA buffer (protease/phosphatase inhibitors), clarified at 12,000 × g, and quantified by BCA. Equal protein (20–40 µg) was resolved by SDS–PAGE and transferred to PVDF. Membranes were blocked in 5% milk and incubated overnight at 4 °C with primary antibodies against PPIP5K2, RBM23, IGF2BP2, TGF-β, COL-I and α-SMA, followed by HRP-secondary antibodies. Bands were visualized by ECL and quantified in ImageJ; targets were normalized to GAPDH.

### Histology and fibrosis quantification

Fixed hearts were paraffin-embedded and sectioned at 4 µm. H&E staining evaluated myocyte disarray, vacuolization, and interstitial changes. Masson’s trichrome visualized collagen deposition. Fibrosis burden was expressed as collagen-positive area (% of total tissue) from ≥ 5 fields/heart (20 ×), quantified by color thresholding in ImageJ by a blinded assessor.

### Statistical analysis

Unless otherwise stated, data are presented as mean ± SEM. Normality was assessed by Shapiro–Wilk. Two-group comparisons used two-tailed Student’s *t*-test or Mann–Whitney *U* as appropriate. Multi-group comparisons (G1–G4) used one-way ANOVA with Tukey’s post hoc test or Kruskal–Wallis with Dunn’s correction. Correlations used Spearman’s *ρ*. For high-dimensional analyses (DEG, enrichment, CIBERSORT cell-type panel), multiple testing was controlled by BH FDR. A *P* < 0.05 (two-sided) denoted statistical significance. All computational analyses were conducted in R (v4.x) with packages limma/edgeR/voom, sva, clusterProfiler, WGCNA, pROC, fgsea, and plotting with ggplot2/pheatmap; docking used AutoDock Vina and PyMOL. Sample sizes, exact n, and any exclusions are specified in the figure legends or Supplementary Methods.

## Results

### Differential expression and functional enrichment delineate proteostasis and metabolism as dominant axes of diabetic cardiac dysfunction-related transcriptomic signatures

**Fig. 1:**
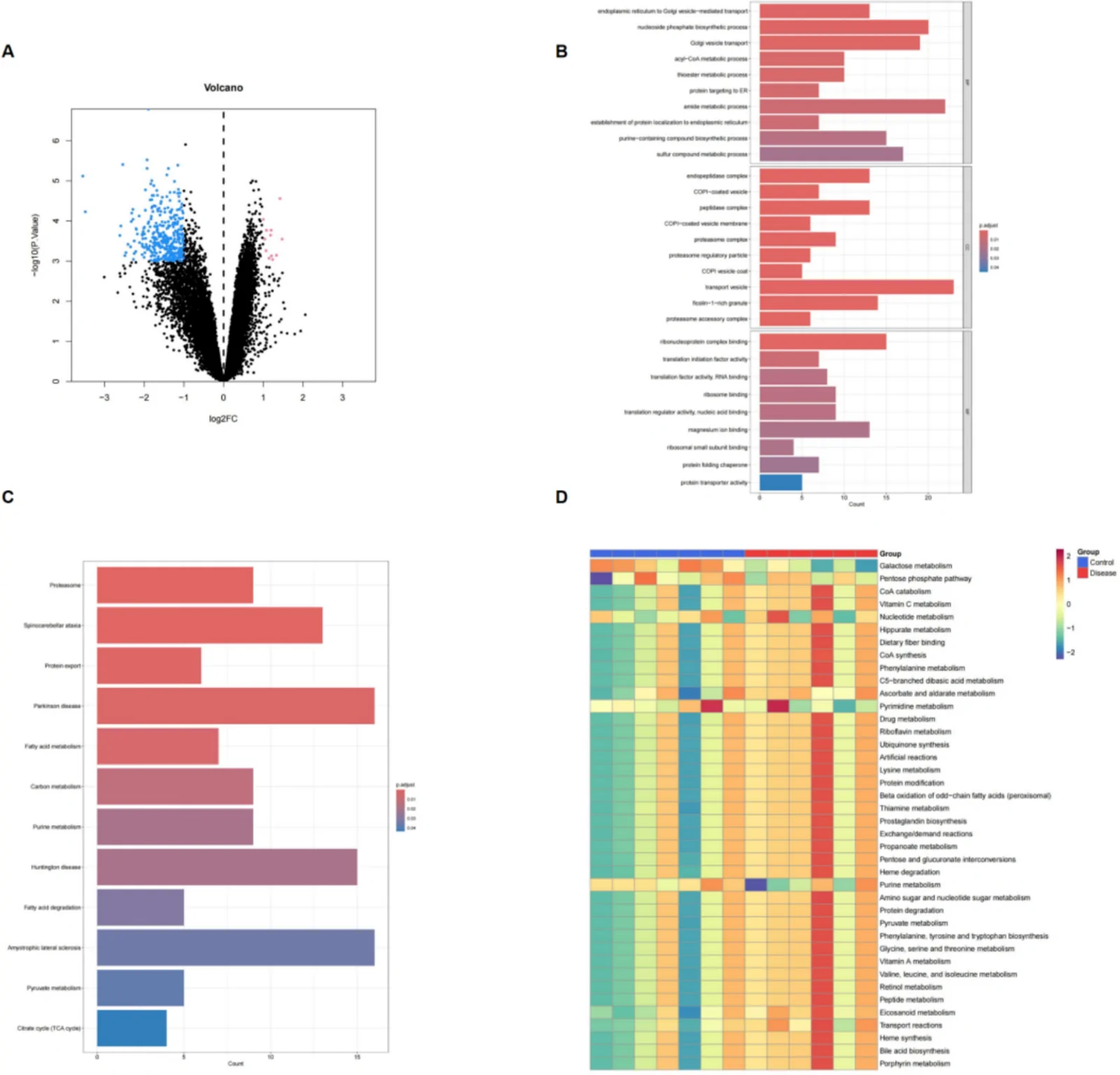
Transcriptomic signatures in diabetic cardiac dysfunction: DEGs and functional landscape. **A** Volcano plot of differential expression between diabetic cardiac dysfunction and control samples (integrated GEO analysis: GSE288708, GSE197850). **B** Gene ontology enrichment of DEGs. The bars depict the top enriched biological process (BP), cellular component (CC), and molecular function (MF) terms ranked by adjusted *P*-value; the bar length indicates gene counts and color encodes FDR. **C** KEGG pathway enrichment of DEGs. Bar length indicates gene counts; color encodes FDR. **D** Heatmap of metabolism pathway activity scores, separating disease/control groups

Analysis of GEO datasets related to diabetic cardiac dysfunction (e.g., GSE288708 and GSE197850) revealed broad transcriptional reprogramming, with a larger proportion of downregulated than upregulated genes (Fig. 1A). GO enrichment showed coordinated changes in processes linking secretory trafficking, proteostasis, and intermediary metabolism. Biological-process terms involved ER–Golgi transport and acyl-CoA/nucleotide-phosphate metabolism; cellular-component terms highlighted COPI-coated vesicles and proteasome complexes; and molecular-function terms involved RNA-binding and chaperone activity (Fig. 1B). KEGG pathway analysis highlighted the proteasome and several core bioenergetic pathways, including fatty acid metabolism and degradation, carbon and purine metabolism, pyruvate metabolism, and the tricarboxylic-acid cycle (Fig. 1C). Consistently, a metabolism-focused heatmap showed coherent separation between groups across carbohydrate (galactose, pentose-phosphate), lipid β-oxidation, amino-acid and vitamin-cofactor programs, as well as redox-linked modules such as ubiquinone and heme/porphyrin turnover (Fig. 1D). Together, these findings define a discovery-layer signature in which impaired ER–Golgi trafficking and heightened protein-quality control intersect with rewired lipid utilization and mitochondrial bioenergetics, providing a mechanistic substrate for downstream network, T2DM-associated MR prioritization, and therapeutic analyses.

Metabolic flux remodeling and co-expression modules converge on a compact candidate set. Using the METAFlux framework (Lu et al. 2022). We first inferred pathway-level fluxes and metabolite activities from the comorbidity-associated gene set. The disease group displayed a coherent reprogramming of central carbon and lipid metabolism: activities linked to β-oxidation, the tricarboxylic-acid cycle and CoA-dependent catabolism were attenuated, whereas pentose-phosphate/cofactor-generating programs and several redox-linked routes (e.g., ubiquinone and heme/porphyrin synthesis) were relatively accentuated. These flux signatures recapitulate the DEG-based enrichment patterns, indicating a shift from efficient mitochondrial bioenergetics toward compensatory, biosynthesis-leaning and oxidative stress–coupled states.

**Fig. 2:**
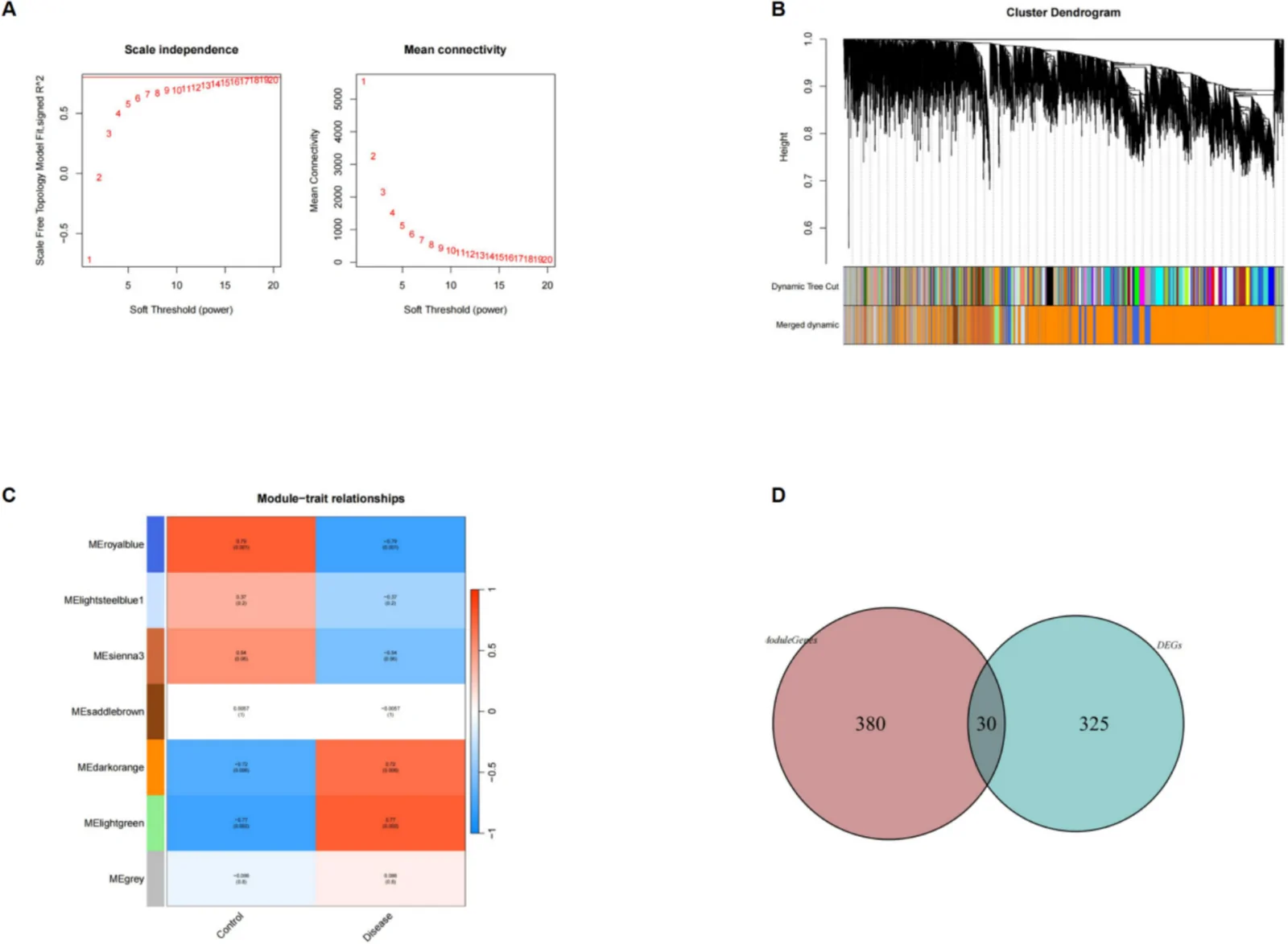
WGCNA workflow and convergence with differential expression. **A** Scale-free topology fit (signed *R*^2^) and mean connectivity across soft-thresholding powers; a value within the plateau region was selected. **B** Gene clustering dendrogram with dynamic tree cut and merged module colors. **C** Module–trait heatmap showing eigengene correlations with control and disease; numbers indicate correlation (top) and *P*-value (bottom). Disease-associated modules include MEdarkorange and MElightgreen (positive) and MEroyalblue/MEsienna3 (negative). **D** Venn diagram of module genes versus DEGs, yielding 30 overlapping candidate genes

To reduce the DEG list to network-level signals associated with phenotype, we constructed a weighted gene co-expression network. Increasing the soft-thresholding power improved the scale-free topology fit (signed *R*^2^ approaching the plateau) while decreasing mean connectivity, and a power within this plateau was chosen to balance both properties (Fig. 2A). Dynamic tree cutting and module merging yielded multiple co-expression modules, with the gray module representing unassigned genes (Fig. 2B). Module–trait analysis revealed reproducible associations with disease status (Fig. 2C): MEdarkorange and MElightgreen were positively correlated with disease, whereas MEroyalblu and MEsienna3 were depleted in cases; other modules showed weak or null correlations. Intersecting genes from the disease-associated modules with the DEG catalog produced a compact set of 30 candidates for downstream genetic prioritization and translational analyses (Fig. 2D). Together, the flux-level perturbations and network-level co-expression signals nominate a focused panel of genes that couple impaired mitochondrial lipid utilization to heightened proteostasis/oxidative stress, providing a biologically plausible link to diabetic cardiac dysfunction-related phenotypes and a tractable entry point for MR and experimental follow-up.

### Mendelian randomization prioritizes expression-associated genes (eGenes) with putative causal effects on T2DM risk

**Fig. 3:**
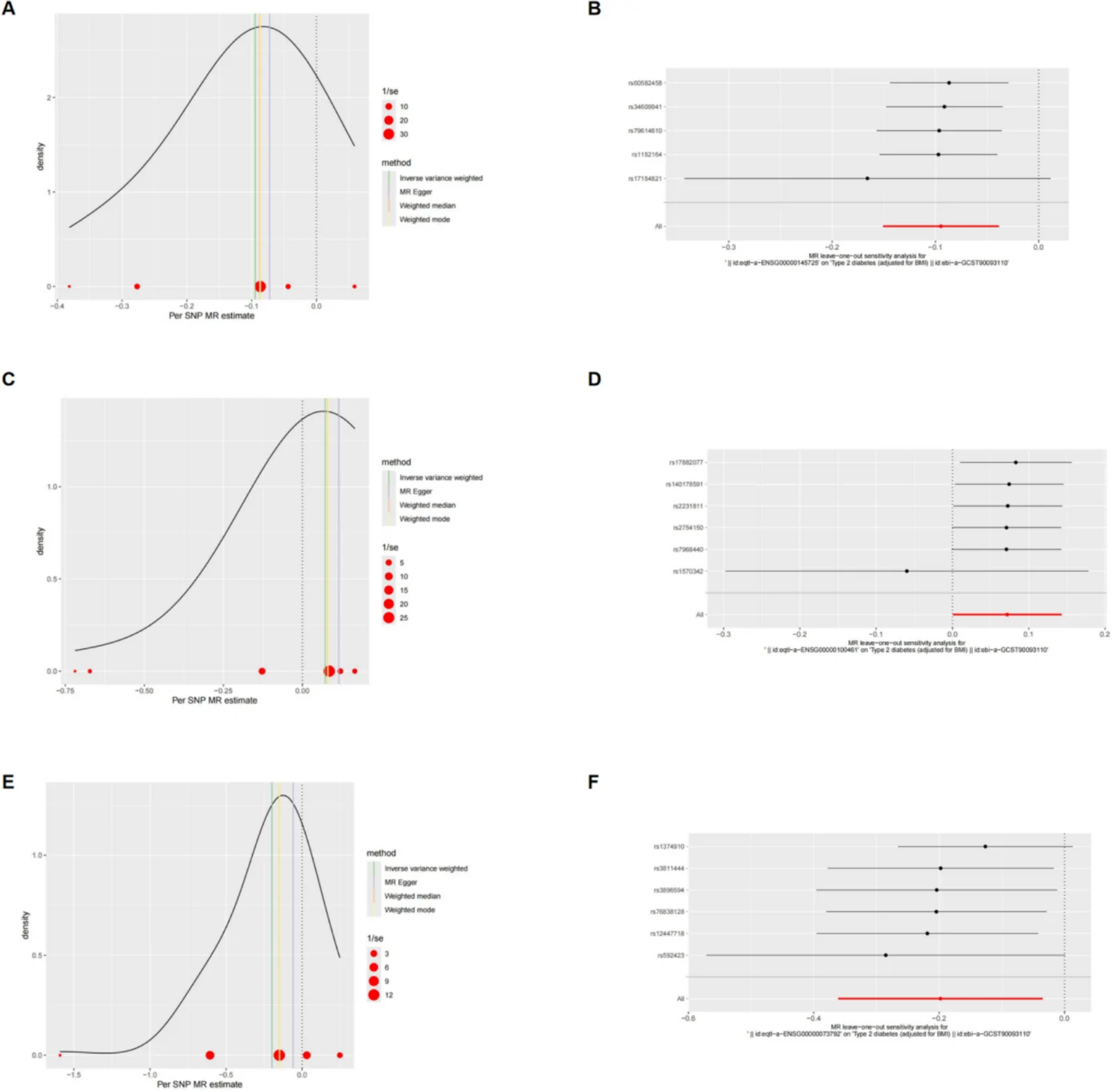
Two-sample MR of eGene expression on BMI-adjusted type 2 diabetes risk. **A**, **C**, and **E** Per-SNP MR estimates with kernel density overlays. Vertical lines mark point estimates from four MR models: inverse-variance weighted (IVW), MR-Egger, weighted median, and weighted mode; the dotted line denotes the null. Panels correspond to ENSG00000145279 (**A**), ENSG00000100461 (**C**), and ENSG00000073792 (**E**). Leave-one-out sensitivity analyses for the corresponding loci; each point shows the IVW estimate with one instrument removed, and the red bar indicates the all-in estimate (**B**, **D**, and **F**). Consistent directions across models and stability in leave-one-out analyses support robustness of the inferred causal effects on T2DM risk

To elevate network-derived candidates to T2DM-associated genetically supported genes, we instrumented gene expression with cis-eQTLs and performed two-sample Mendelian randomization (MR) against BMI-adjusted type 2 diabetes risk. Across the 30 intersecting genes, three eGenes displayed directionally consistent and method-concordant effects (Fig. 3A–F). For ENSG00000145279, the inverse-variance–weighted (IVW) estimate indicated a protective association of higher genetically proxied expression with T2D, closely mirrored by MR-Egger, weighted median, and weighted mode estimators (Fig. 3A). Leave-one-out analyses showed that omitting any single instrument did not materially shift the overall effect (Fig. 3B), arguing against domination by a single variant. ENSG00000100461 showed the opposite pattern, with a positive association between higher expression and agreement across sensitivity estimators (Fig. 3C); the leave-one-out profile again suggested robustness to variant omission (Fig. 3D). A second inverse signal was observed at ENSG00000073792, where all four MR estimators clustered on the protective side of the null, and leave-one-out curves retained the negative direction throughout (Fig. 3E–F). Taken together, these results nominate a short list of expression-based T2DM-associated genetically supported candidates, including two genes showing protective associations and one gene showing a risk-enhancing association with T2D, thereby providing genetically anchored targets for downstream mechanistic studies and therapeutic evaluation.

### External validation in GEO cohorts and diagnostic discrimination of T2DM-associated key genes

**Fig. 4:**
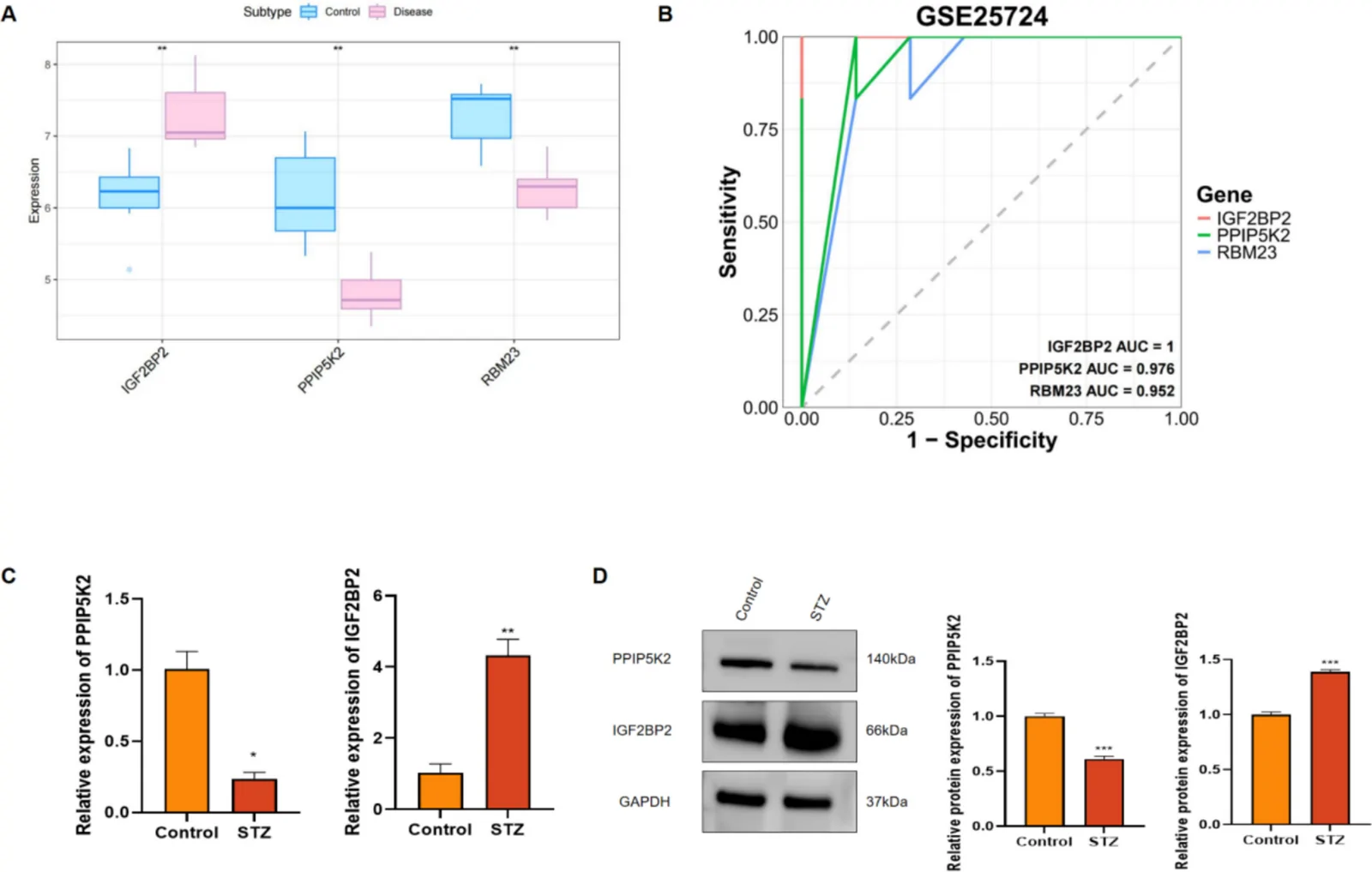
Multi-level validation of T2DM-associated key genes in human cohorts and STZ-induced diabetic rats. **A** Expressions of IGF2BP2, PPIP5K2, and RBM23 in training data, stratified by subtype (blue, control; pink, disease). IGF2BP2 is upregulated in disease, while PPIP5K2 and RBM23 are downregulated. **B** Receiver-operating characteristic (ROC) curves in the validation cohort (GSE25724), showing AUCs of 1.00 (IGF2BP2), 0.976 (PPIP5K2), and 0.952 (RBM23). Additional summary panels (if shown) report concordant performance across training and validation sets. **C** Validation of gene expression in the STZ-induced diabetic rat model by qRT-PCR. PPIP5K2 expression is decreased in diabetic rats, whereas IGF2BP2 expression is increased, consistent with findings in human datasets. **D** Protein expression of PPIP5K2 and IGF2BP2 in rat myocardium assessed by Western blotting. Representative blots and quantitative densitometry show reduced PPIP5K2 and increased IGF2BP2 protein levels in STZ-treated rats compared with controls

We next evaluated whether the MR-prioritized candidates show reproducible case–control differences and diagnostic value in independent GEO cohorts. In the training cohort, IGF2BP2 expression was significantly higher in disease than in controls, whereas PPIP5K2 and RBM23 were lower in disease (box-and-whisker plots, Fig. 4A). The directionality mirrors the discovery layer and is consistent with the genetic inference of risk-enhancing versus protective effects. In an external validation set (GSE25724), all three markers exhibited strong discrimination of cases from controls (ROC curves, Fig. 4B), with AUC = 1.00 for IGF2BP2, 0.976 for PPIP5K2, and 0.952 for RBM23. Together, these results indicate that the shortlisted transcripts are consistently dysregulated in diabetic cardiac dysfunction-related cohorts and each gene showed high diagnostic performance across datasets. Given the heterogeneity of the source cohorts, this diagnostic performance should be interpreted as cohort-level discrimination and requires further validation in clinically well-characterized DCM cohorts.

To further corroborate these findings in vivo, we measured myocardial mRNA and protein levels of IGF2BP2 and PPIP5K2 in STZ-induced diabetic rats. qRT-PCR showed increased IGF2BP2 and decreased PPIP5K2 expression in diabetic hearts compared with controls (Fig. 4C), and Western blotting demonstrated the same directional changes at the protein level (Fig. 4D). Together, these results indicate that the shortlisted transcripts are consistently dysregulated in diabetic cardiac dysfunction across human cohorts and the STZ model, and that each gene shows potential diagnostic or translational relevance that warrants further validation.

### Immune infiltration and single-gene GSEA link key transcripts to innate activation and proteostasis–metabolic programs

**Fig. 5:**
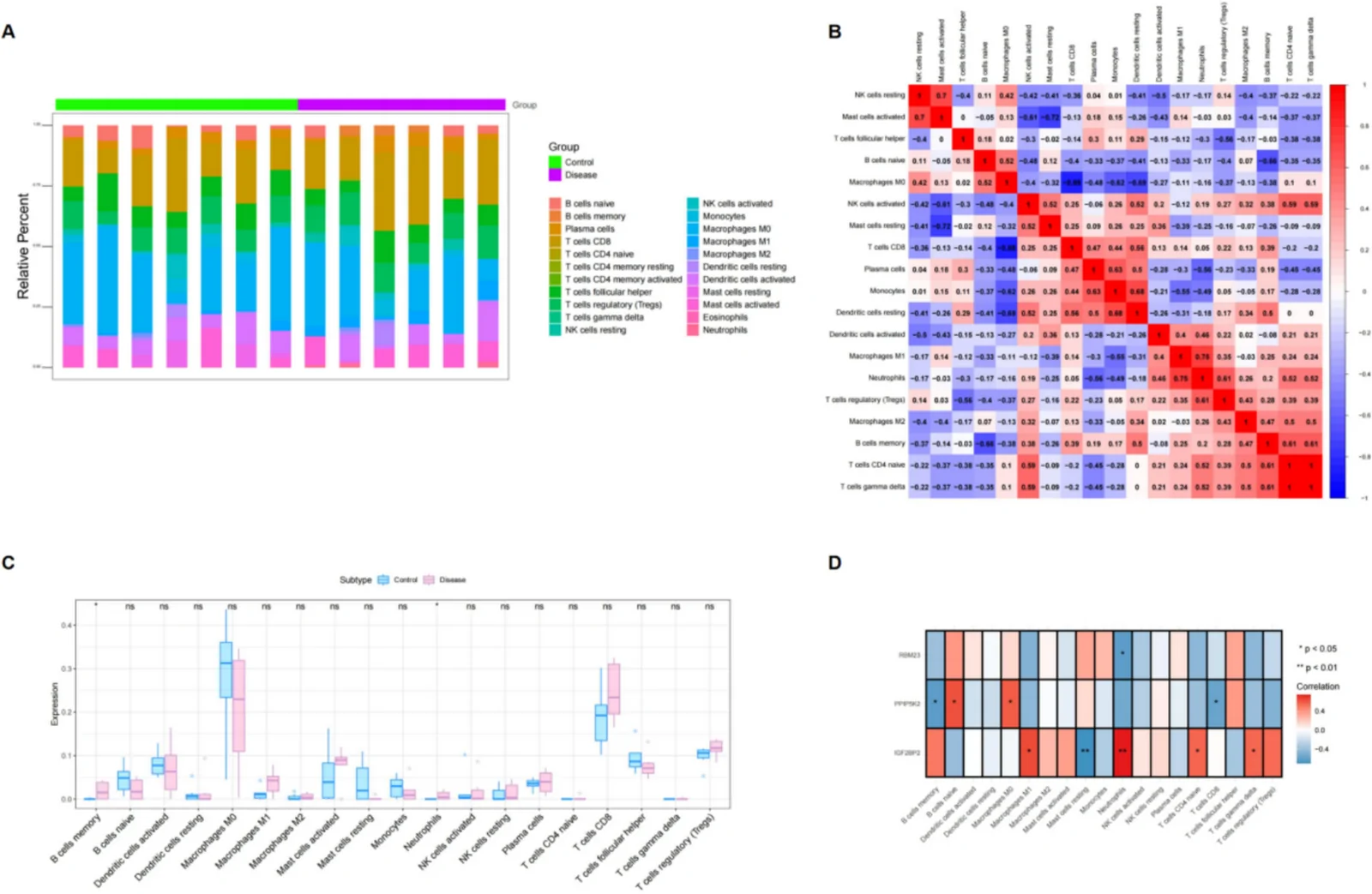
Immune infiltration (CIBERSORT) and gene–immune coupling. **A** Stacked bar plot of relative leukocyte fractions across samples, grouped by case–control status. **B** Pairwise correlation matrix for the 22 immune cell types (Spearman’s *ρ*), highlighting coordinated innate and adaptive modules. **C** Box-and-whisker comparisons of cell-type fractions between groups; significance annotations are shown above each facet. **D** Heatmap of correlations between key transcripts (IGF2BP2, PPIP5K2, RBM23) and immune subsets; asterisks denote FDR-adjusted significance (*P* < 0.05; *P < 0.01)

CIBERSORT deconvolution revealed a reproducible reshaping of the immune milieu between cases and controls. At the compositional level, disease samples showed a higher contribution from macrophage compartments, with M0 appearing most increased and a possible shift toward M1 polarization, accompanied by modest increases in follicular helper T cells and plasma cells, while resting NK cells were relatively depleted (Fig. 5A). The global correlation structure among the 22 leukocyte subsets recapitulated known antagonisms (e.g., inverse relationships between M0/M1 macrophages and cytotoxic or NK lineages) and positive couplings within innate modules (monocytes–macrophages) and adaptive modules (B-cell memory–Tfh), indicating coordinated remodeling rather than isolated fluctuations (Fig. 5B). Groupwise comparisons highlighted modest but directionally consistent shifts, with resting NK cells reduced and macrophage M0/M1 fractions increased in disease, while several other subsets varied little across groups (Fig. 5C).

Correlation analysis showed that the three MR-prioritized genes exhibited distinct immune-association patterns (Fig. 5D). RBM23 displayed a negative correlation with neutrophils. PPIP5K2 showed negative correlations with B-cell memory and CD8⁺ T cells, while exhibiting positive associations with B-cell naive subsets and macrophages M0. IGF2BP2 was positively correlated with macrophages M1, neutrophils, CD4⁺ naive T cells, and γδ T cells, and negatively correlated with resting mast cells. These patterns suggest that each candidate gene is linked to specific components of the cardiac immune microenvironment. Together, these findings position the shortlisted genes at the interface of innate immune activation and tissue remodeling, consistent with the proteostasis and metabolic axes identified in our enrichment analyses. However, these immune associations should be viewed as correlative transcriptomic evidence and require functional validation. Single-gene GSEA further supported this view: higher IGF2BP2 was enriched for RNA handling/translation, proteasome and inflammatory signaling; higher PPIP5K2 aligned with ER–Golgi trafficking, vesicle transport and lipid/CoA metabolism; higher RBM23 mapped to RNA processing and mitochondrial oxidative phosphorylation, reinforcing a mechanistic bridge between immune tone, protein quality control and myocardial bioenergetics.

### Network-guided drug repurposing nominates the sesquiterpene lactone cynaropicrin; docking supports hypothesis-generating interaction models

**Fig. 6:**
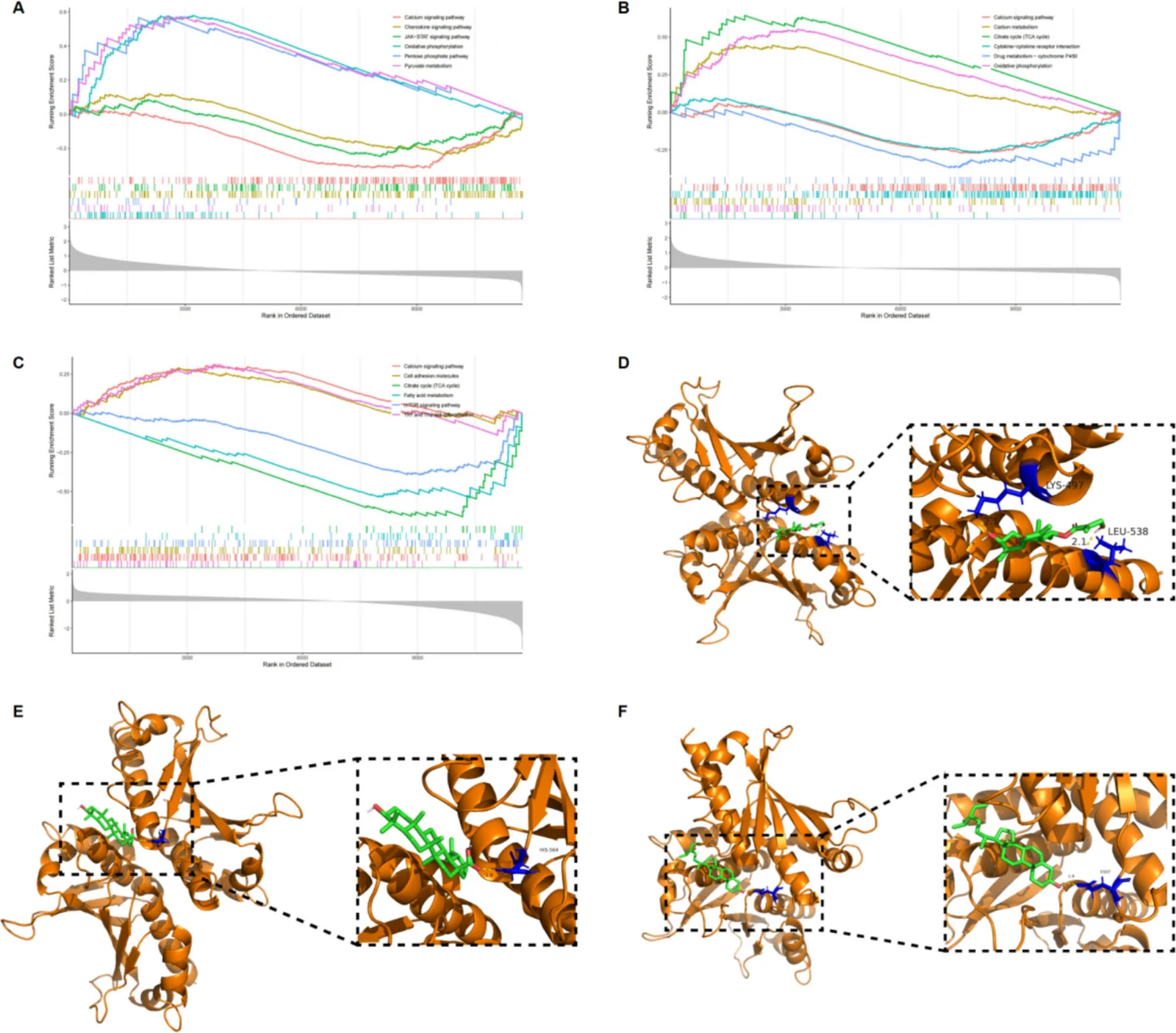
Reverse-transcriptome prioritization, single-gene GSEA, and docking of cynaropicrin. **A**–**C** Single-gene GSEA for IGF2BP2 (**A**), PPIP5K2 (**B**), and RBM23 (**C**). Enrichment plots show coordinated positive enrichment of calcium signaling/cardiac contraction and oxidative phosphorylation/TCA cycle, with cardiomyopathy-related gene sets changing concordantly. Tick marks indicate gene set hits along the ranked transcriptome; the running enrichment score and leading-edge subsets are displayed as standard. **D**–**F** Representative docking poses of cynaropicrin (green sticks) within pockets of the three proteins (cartoon). Insets highlight hydrogen-bond and hydrophobic contacts that stabilize the complexes; dashed lines denote key interactions, with contact distances on the order of 2–3 Å. These models suggest computationally feasible interaction poses between cynaropicrin and the candidate proteins and motivated its selection for STZ-induced diabetic rat experiments

To translate the transcriptomic/causal signals into pharmacologic hypotheses, we queried CTDbase, DSigDB, and Coremine Medical with the disease signature and the three MR-prioritized genes (PPIP5K2, RBM23, IGF2BP2). Convergent hits across libraries were enriched for natural products with anti-inflammatory and proteostasis-modulating properties, among which the sesquiterpene lactone cynaropicrin emerged as a consistent candidate for downstream validation. In parallel, single-gene GSEA anchored the biology of each key transcript to drug-tractable pathways (Fig. 6A–C): samples stratified by IGF2BP2, PPIP5K2, or RBM23 showed coordinated enrichment of calcium handling and cardiac muscle contraction, together with oxidative phosphorylation and the tricarboxylic-acid cycle (with cardiomyopathy-linked sets tracking in the same direction), supporting a potential link between proteostasis/translation control and mitochondrial bioenergetics.

Molecular docking of cynaropicrin against structural models of the three proteins yielded computationally favorable poses within well-formed pockets (Fig. 6D–F). The predicted complexes are stabilized by hydrogen bonds and hydrophobic packing around canonical clefts, placing the lactone ring in proximity to polar residues and the hydrophobic scaffold against non-polar side chains, with contact distances in the ~ 2 to 3 Å range. Although exploratory, these interactions are chemically plausible for a Michael-acceptor-bearing scaffold and align with the pathway context highlighted by GSEA. On the strength of (i) cross-database prioritization, (ii) coherence with the proteostasis–metabolic axis identified in our omics analyses, and (iii) hypothesis-generating docking evidence, we selected cynaropicrin for in vivo validation.

### Integrative transcriptomics and Mendelian randomization identify T2DM-associated candidate genes and support cynaropicrin as a candidate intervention in diabetic cardiac injury

**Fig. 7:**
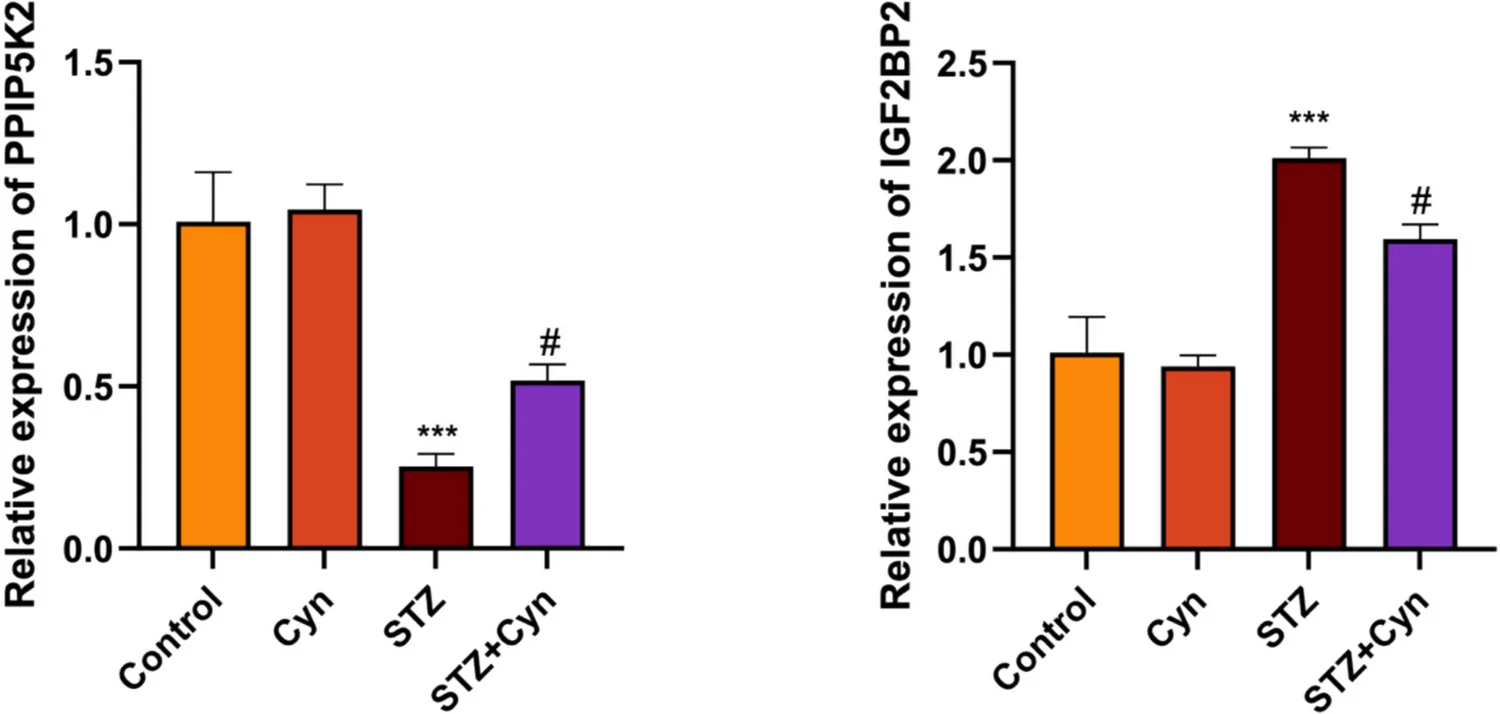
qRT–PCR validation of key transcripts in the diabetic myocardium. Relative mRNA abundance of PPIP5K2 and IGF2BP2 in left-ventricular tissue from control and STZ-treated rats

In the high-fat diet plus streptozotocin (STZ) model, myocardial expression of the three MR-prioritized transcripts mirrored the human discovery layer. Quantitative RT–PCR showed IGF2BP2 to be upregulated in diabetic hearts relative to controls, whereas PPIP5K2 and RBM23 were downregulated. The directionality is concordant with the GEO validation and with the inferred risk-enhancing (IGF2BP2) versus protective (PPIP5K2/RBM23) effects on T2DM risk from the genetic analyses, consolidating these transcripts as disease-associated readouts in vivo (Fig. 7). These data support their relevance to diabetic myocardial injury and remodeling-related phenotypes.

**Fig. 8:**
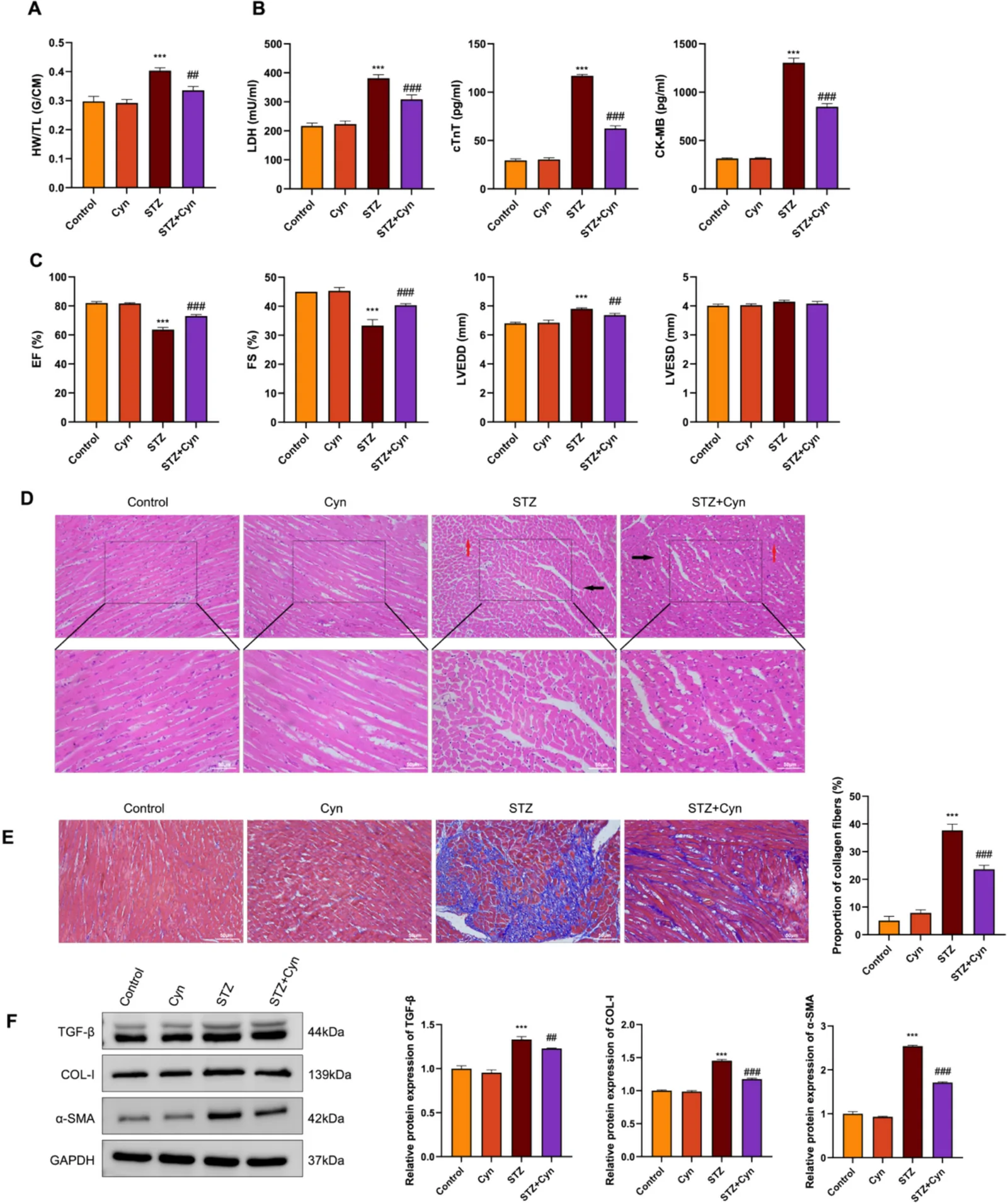
Cynaropicrin mitigates cardiac injury and remodeling in STZ-induced diabetes. **A** Cardiac hypertrophy indexed by heart weight/tibia length (HW/TL). STZ increased HW/TL versus Control; cynaropicrin reduced HW/TL in diabetic rats toward baseline, with no change in non-diabetic Cyn versus Control. **B** Serum injury biomarkers (LDH, cTnT, CK-MB). Levels rose in STZ and were attenuated in STZ + Cyn; Cyn alone remained comparable to Control. **C** Echocardiography. Diabetic rats developed systolic dysfunction (lower EF and FS) and chamber dilatation (higher LVEDD and LVESD). Cynaropicrin improved EF/FS and limited LV dilation relative to STZ. **D** H&E histology of left ventricle; STZ hearts show myofiber disarray, cytoplasmic vacuolization, and interstitial expansion (arrows); these lesions are visibly mitigated in STZ + Cyn. Representative fields are shown; scale bars as indicated. **E** Masson’s trichrome staining. Interstitial/perivascular collagen (blue) is increased in STZ and reduced by cynaropicrin; the right panel quantifies fibrotic area (% of tissue). **F** Western blot and densitometry for profibrotic markers (TGF-β, COL-I, α-SMA) in ventricular tissue. STZ elevates all three; cynaropicrin suppresses their expression toward Control

To evaluate the in vivo cardiometabolic effects and therapeutic plausibility of cynaropicrin, we randomized rats to four 8-week regimens—Control, Cyn, STZ, and STZ + Cyn. As anticipated, diabetic animals developed a classic cardiomyopathic phenotype with cardiac hypertrophy (higher heart weight/tibia length ratio, HW/TL; Fig. 8A), biochemical evidence of myocyte injury, and systolic dysfunction accompanied by ventricular dilation (Fig. 8C). Histology corroborated these changes: hematoxylin–eosin sections revealed myofiber disarray, cytoplasmic vacuolization, and interstitial expansion in STZ hearts, whereas these lesions were visibly mitigated when cynaropicrin was co-administered (Fig. 8D). Masson’s trichrome staining further showed expanded interstitial/perivascular collagen in STZ that was reduced by cynaropicrin, with a corresponding decrease in the fibrotic area percentage on quantitative analysis (Fig. 8E). At the signaling level, immunoblotting documented activation of a pro-fibrotic program in diabetic myocardium, with TGF-β, COL-I, and α-SMA all increased, and these elevations were suppressed toward baseline in the STZ + Cyn arm (Fig. 8F). Importantly, cynaropicrin administered to non-diabetic rats did not materially alter structural, functional, histological, or molecular readouts relative to controls across panels A–F, indicating disease-selective benefit. Collectively, Fig. 8A–F demonstrates that cynaropicrin attenuates hypertrophy and biochemical injury, improves contractile performance while limiting chamber dilation, reduces histopathological damage and collagen accumulation, and down-modulates canonical TGF-β/activated-myofibroblast signaling in diabetic hearts.

**Fig. 9:**
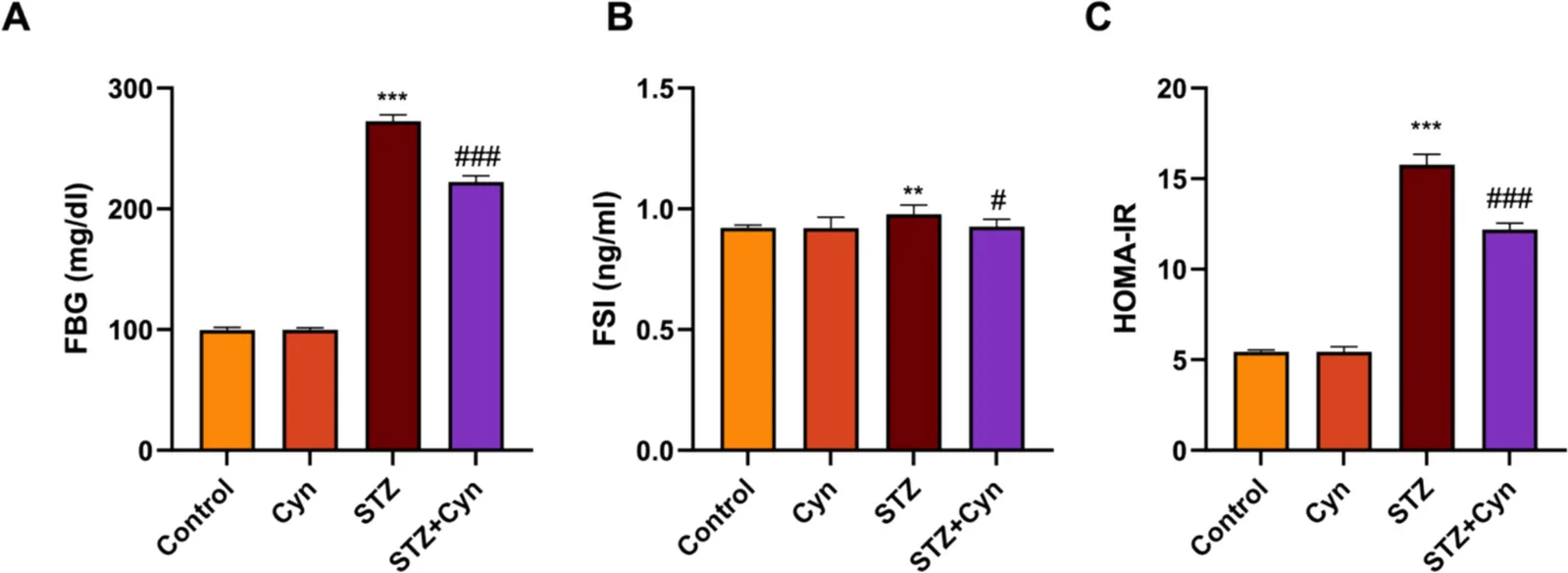
Cynaropicrin improves glycemic control and insulin resistance. **A** Fasting blood glucose (FBG); STZ elevates FBG; cynaropicrin lowers FBG in diabetic rats toward control levels, with little change in Cyn versus Control. **B** Fasting serum insulin (FSI); STZ perturbs FSI; cynaropicrin partially restores it toward baseline. **C** HOMA-IR; insulin resistance increases with STZ and is reduced by cynaropicrin in the diabetic arm, with negligible effects in non-diabetic rats

To determine whether the cardioprotective effects were accompanied by systemic metabolic benefits, we quantified fasting blood glucose (FBG), fasting serum insulin (FSI), and the homeostatic model assessment of insulin resistance (HOMA-IR) after 8 weeks of treatment. STZ administration produced the expected metabolic derangements, with marked fasting hyperglycemia and elevated HOMA-IR relative to non-diabetic controls, indicating pronounced insulin resistance. Cynaropicrin significantly lowered FBG and reduced HOMA-IR in diabetic animals, shifting values toward the control range, while exerting minimal effects in non-diabetic rats. FSI was perturbed by STZ, consistent with β-cell dysfunction/compensatory drive, and cynaropicrin partially normalized this parameter. Collectively, these data show that cynaropicrin ameliorates the diabetic milieu in parallel with its direct cardiac benefits (Fig. 9A–C). Therefore, part of the observed myocardial improvement may be associated with systemic metabolic improvement, and direct myocardial target engagement requires further investigation.

**Fig. 10:**
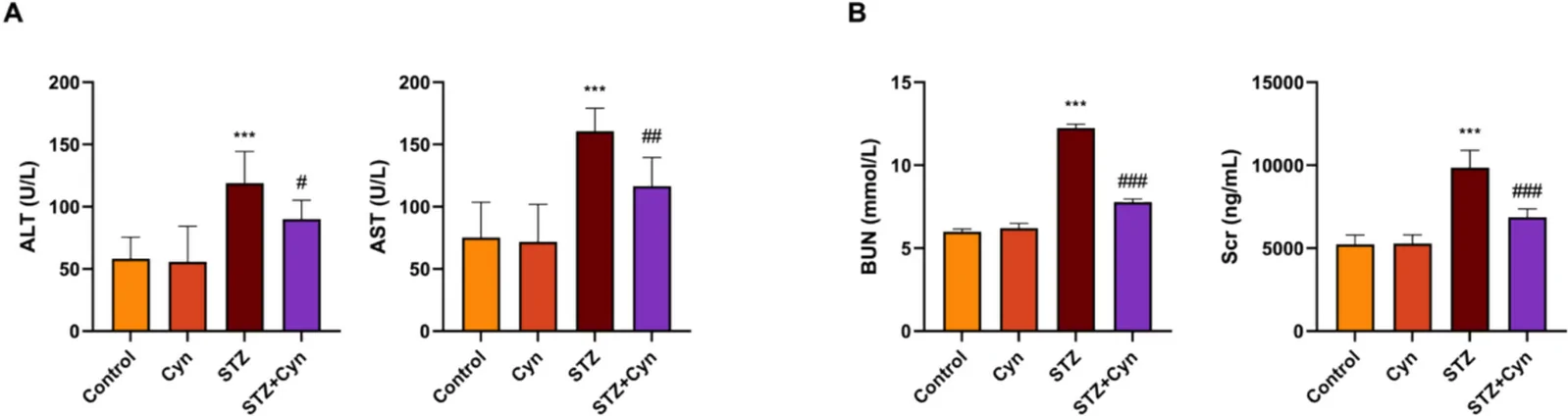
Hepatic and renal function after 8 weeks of treatment: **A** Serum ALT and AST across control, Cyn, STZ, and STZ + Cyn groups and **B** BUN and serum creatinine (Scr) in the same four groups

To extend safety and organ-system readouts beyond the heart, we quantified canonical liver enzymes and renal indices after 8 weeks of treatment. Diabetic rats exhibited a clear biochemical injury pattern, with ALT and AST elevated relative to non-diabetic controls and accompanied by increases in BUN and serum creatinine (Scr), which is consistent with diabetes-associated hepatorenal stress.. Cynaropicrin co-administration (STZ + Cyn) shifted all four markers toward baseline, lowering transaminases and renal indices compared with STZ alone, whereas cynaropicrin in non-diabetic animals (Cyn) remained indistinguishable from Control across measurements. These findings indicate that cynaropicrin did not produce obvious hepatic or renal biochemical toxicity under the present experimental conditions and may confer hepatoprotective and renoprotective effects in the diabetic milieu, complementing its cardioprotective actions (Fig. 10A–B).

## Discussion

This study integrates transcriptomic analysis and Mendelian randomization to identify biomarkers associated with diabetic cardiac dysfunction-related transcriptomic signatures and genetically supported T2DM risk, culminating in the experimental validation of cynaropicrin as a candidate cardiometabolic intervention. Our multi-dimensional approach has yielded several key findings that may advance understanding of diabetes-associated myocardial injury and remodeling-related biology and provide translational insights for precision medicine.

Our transcriptomic analysis uncovered a predominant downregulation pattern in diabetic cardiac dysfunction-related datasets, with functional enrichment converging on proteostasis disruption and metabolic reprogramming. The emphasis on ER–Golgi trafficking, proteasome function, and mitochondrial bioenergetics aligns with emerging concepts of diabetic cardiomyopathy as a disorder of cellular quality control systems (Aragam and Natarajan 2020). These findings parallel recent single-cell studies of diabetic hearts, which have identified similar perturbations in cardiomyocyte proteostasis and metabolic flexibility (McDonagh et al. 2022). The coherent separation of metabolic pathway activities between disease and control groups, particularly involving fatty acid β-oxidation and tricarboxylic acid cycle components, supports the established paradigm of cardiac metabolic inflexibility in diabetes (Mileo et al. 2019).

Our WGCNA analysis successfully reduced the complexity of transcriptomic data to focused co-expression modules strongly associated with disease phenotype. The identification of 30 candidate genes from the intersection of differentially expressed genes and disease-associated modules represents a substantial refinement from genome-wide signals, demonstrating the power of network-based approaches in biomarker discovery (Parim et al. 2019). This methodology has proven effective in similar studies of diabetic complications, where co-expression networks have revealed previously unrecognized disease-associated modules (Bowden et al. 2015; Conning-Rowland et al. 2024; Cui et al. 2024a).

The application of two-sample Mendelian randomization to our network-derived candidates represents a critical advancement in moving beyond mere association to genetic prioritization. Our MR analysis suggests potential causal relationships of PPIP5K2, RBM23, and IGF2BP2 with T2DM risk, helping address limitations of observational transcriptomic studies. Accordingly, these genes are best described as T2DM-associated genetically supported candidates in the present study. The directional consistency across multiple MR estimators (IVW, MR-Egger, weighted median, and weighted mode) and robust leave-one-out analyses strengthen confidence in these genetic inferences (Pushpakom et al. 2019).

The protective effects of higher PPIP5K2 expression, contrasted with the risk-enhancing effects of IGF2BP2, align with their known biological functions. PPIP5K2, involved in inositol phosphate signaling, has been implicated in metabolic homeostasis and insulin sensitivity (Rangboo et al. 2016). RBM23, as an RNA-binding protein, plays crucial roles in post-transcriptional regulation that may influence cardiac stress responses (Richardson et al. 2017). IGF2BP2’s association with increased T2DM risk is consistent with its established role in glucose metabolism and β-cell function (Sattar et al. 2023). Recent proteome-wide MR studies have similarly identified RNA-binding proteins and metabolic regulators as genetically supported factors in cardiometabolic diseases, supporting the biological plausibility of our findings (Frangogiannis 2021). Further cardiac phenotype-specific genetic analyses and functional perturbation experiments are warranted to clarify their mechanistic roles in diabetic myocardial remodeling.

The biomarker panel demonstrated strong diagnostic performance in independent GEO cohorts (AUCs: IGF2BP2 = 1.00, PPIP5K2 = 0.976, RBM23 = 0.952), indicating promising cohort-level discrimination. However, because the included datasets may differ in cardiac phenotype, tissue source, disease duration, treatment exposure, and comorbid heart failure status, these findings require validation in clinically well-characterized diabetic cardiomyopathy cohorts. The consistent directionality of expression changes across discovery and validation cohorts further supports the biological relevance of these targets.

Our immune infiltration analysis revealed coordinated remodeling of the cardiac immune microenvironment, with increased macrophage activation and shifts in T-cell populations. PPIP5K2, RBM23, and IGF2BP2 were associated with both metabolic stress and inflammatory remodeling, although functional studies are required to define their mechanistic roles in diabetic hearts (See et al. 2017). These immune associations should therefore be viewed as correlative transcriptomic evidence. This interpretation aligns with recent studies demonstrating the importance of immune-metabolic crosstalk in diabetic cardiomyopathy progression (Aragam and Natarajan 2020; Song et al. 2025).

Our multi-database screening approach successfully nominated cynaropicrin as a candidate compound, demonstrating the value of integrating computational drug discovery with mechanistic insights. The convergent identification of cynaropicrin across CTDbase, DSigDB, and Coremine Medical, combined with favorable molecular docking profiles, provides a rational foundation for experimental validation. However, molecular docking only provides hypothetical structural compatibility and cannot confirm direct binding, target engagement, or pharmacological mechanism. This approach addresses limitations of traditional phenotypic screening by incorporating molecular mechanism-based selection criteria (Travers et al. 2016).

The experimental validation in STZ-induced diabetic rats convincingly demonstrated cynaropicrin’s cardiometabolic protective potential. The compound attenuated cardiac hypertrophy, improved systolic function, reduced fibrosis, and modulated pro-fibrotic signaling pathways (TGF-β, COL-I, α-SMA), suggesting that its benefits may involve multiple biological processes. These effects are consistent with cynaropicrin’s reported anti-inflammatory and anti-fibrotic properties in other disease contexts (Langfelder and Horvath 2008; Larsson et al. 2020). Importantly, the concurrent improvement in glycemic control and insulin sensitivity suggests that cynaropicrin may address both the underlying metabolic dysfunction and its cardiac consequences. This also indicates that the cardiac benefits may be mediated partly through systemic metabolic improvement, in addition to potential direct myocardial effects.

The suppression of TGF-β signaling is particularly noteworthy, as this pathway represents a central hub in cardiac fibrosis development (Huang et al. 2023; Jia et al. 2018). Recent meta-analyses of anti-diabetic agents have highlighted TGF-β modulation as a key mechanism underlying cardioprotective effects, positioning cynaropicrin within an established therapeutic paradigm (Wilson et al. 2013). The concurrent improvements in hepatic and renal biochemical markers, together with the cardiac benefits, suggest that cynaropicrin may confer benefits beyond the myocardium, although its systemic effects require further study.

Biomarker-based risk stratification may enable earlier identification of diabetic patients who are at higher risk of cardiac dysfunction or remodeling-related phenotypes, facilitating personalized monitoring and timely intervention to prevent progression to overt heart failure (Yang et al. 2025). The integration of genetic risk scores with transcriptomic biomarkers represents an emerging frontier in precision cardiology (Zayed et al. 2020).

For therapeutic development, cynaropicrin’s natural product origin and reported bioactivity of artichoke-derived compounds provide advantages for translation (Zheng et al. 2018). The compound’s broad biological effects, including improvements in inflammation, fibrosis, and metabolic parameters, suggest therapeutic potential that may extend beyond a single pathway. However, cynaropicrin-specific pharmacokinetics, dose–response relationships, long-term safety, and direct target engagement remain to be systematically established. Future studies should explore optimal dosing regimens, combination therapies, and biomarker-guided treatment strategies.

Several limitations merit consideration. First, our transcriptomic analysis relied on publicly available datasets with potential batch effects and heterogeneity in sample characteristics. More importantly, the disease definitions, tissue sources, cardiac phenotypes, disease duration, treatment background, and comorbid heart failure status were not fully harmonized across datasets. Therefore, the integrated signature may capture diabetic cardiac dysfunction, heart failure, and broader cardiomyopathy-related transcriptional changes. Although we employed robust normalization and batch correction procedures, residual confounding cannot be entirely excluded. Second, the Mendelian randomization analysis was limited to cis-eQTL instruments, potentially missing trans-acting regulatory variants. In addition, the MR outcome was BMI-adjusted T2DM risk; consequently, the MR findings support T2DM-associated genetic prioritization of candidate genes. Third, the experimental validation was conducted in a single animal model, and translation to human diabetic cardiomyopathy requires confirmation in clinical studies. Moreover, the present animal data cannot fully separate direct myocardial effects of cynaropicrin from indirect benefits mediated by improved glycemic control and insulin resistance.

The molecular docking predictions, while supporting hypothetical interaction models, require experimental validation through biochemical binding assays and mechanism-of-action studies. Thus, conclusions regarding direct binding to PPIP5K2, RBM23, or IGF2BP2 should be avoided until such evidence is available. Additionally, the downstream signaling pathways mediating cynaropicrin’s effects warrant detailed investigation to fully understand its therapeutic mechanisms.

This study establishes a novel paradigm for biomarker discovery in diabetic cardiac dysfunction-related research by integrating transcriptomic analysis with Mendelian randomization to identify T2DM-associated genetically supported candidate genes. The identification of PPIP5K2 and IGF2BP2 as validated biomarkers provides new insights into the molecular mechanisms underlying diabetes-related myocardial injury and remodeling phenotypes. RBM23 also emerged as a genetically supported candidate in the MR analysis, but it was not incorporated into the validation experiments owing to the lack of suitable antibodies for reliable detection. The translational potential of these findings, which enabled the nomination and experimental validation of cynaropicrin as a candidate intervention, highlights the value of multi-dimensional approaches in drug discovery. Nevertheless, the present evidence supports cynaropicrin as a promising candidate rather than a confirmed targeted therapy, and further studies are required to define its direct targets and mechanism of action.

## Conclusion

This study integrates transcriptomic analysis with Mendelian randomization to identify PPIP5K2 and IGF2BP2 as biomarkers associated with diabetic cardiac dysfunction-related transcriptomic signatures and genetically supported T2DM risk, with RBM23 emerging as an additional MR-supported candidate requiring further validation. Through network-guided drug discovery, we identified and experimentally validated cynaropicrin as a promising candidate intervention that significantly improves cardiac function, reduces fibrosis, and enhances metabolic control in diabetic rats. These findings establish a robust foundation for clinical translation, offering both a biomarker panel for risk stratification and a promising natural product intervention for diabetes-associated cardiac dysfunction. However, validation in clinically well-defined diabetic cardiomyopathy cohorts and direct target-engagement studies are required before clinical or mechanistic conclusions can be finalized.

## Supplementary Information

Below is the link to the electronic supplementary material.

ESM 1 (PDF 0.98 MB)

ESM 2 (DOCX 11.8 KB)

## Data Availability

All source data for this work (or generated in this study) are available upon reasonable request.
