# Supplementary material for: Integrated transcriptomic and Mendelian randomization analysis identifies novel biomarkers for type 2 diabetes–associated cardiac dysfunction: cynaropicrin as a candidate intervention
Source: Naunyn Schmiedebergs Arch Pharmacol. 2026 Jul 10;399(13):20885–902. doi: 10.1007/s00210-026-05585-2 (PMC13695668; doi:10.1007/s00210-026-05585-2)
Supplement: Supplementary file 1 [file 210_2026_5585_MOESM1_ESM.pdf]

Figure 4D

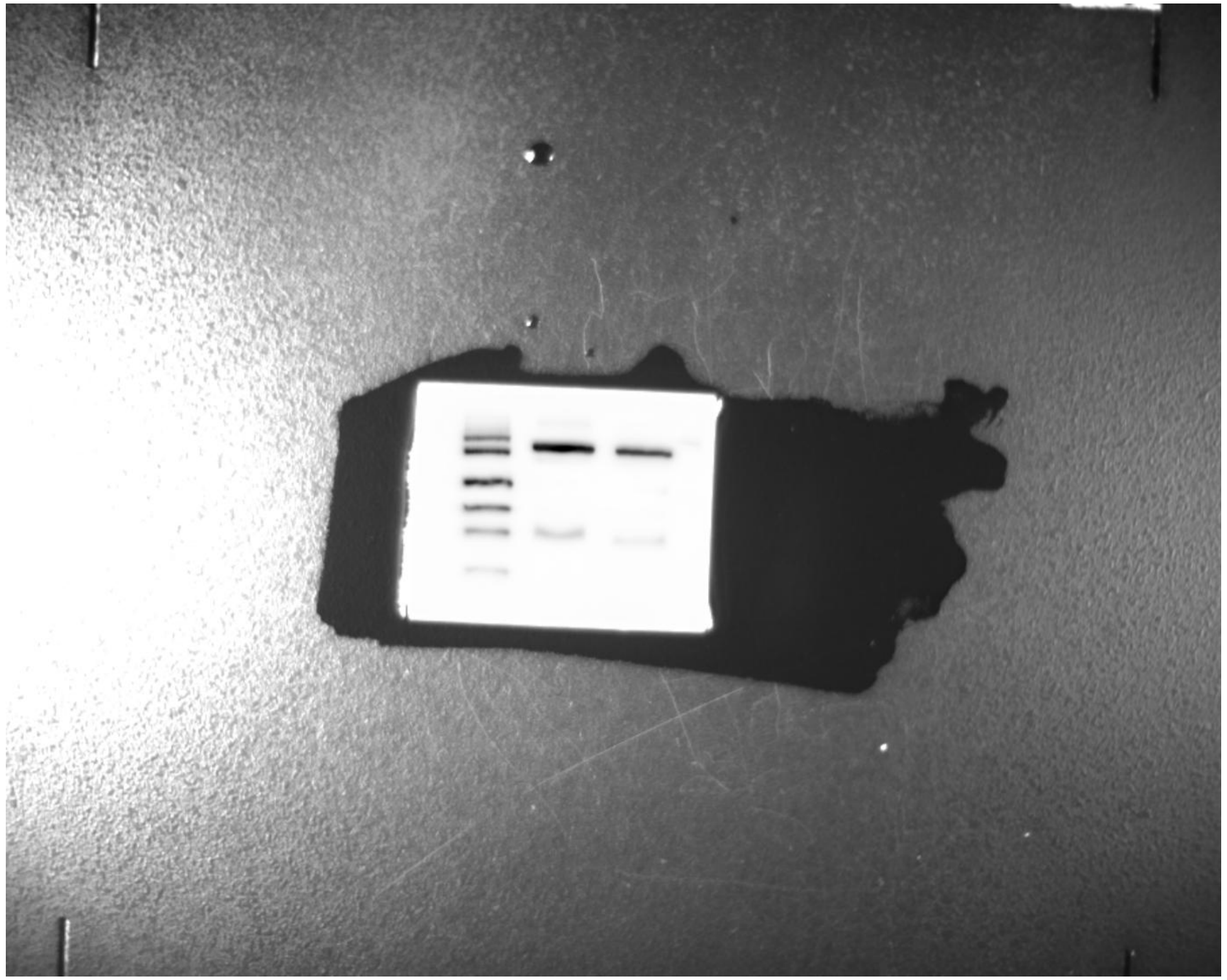

The original Western Blot images of PPIP5K2 in Figure 4D. From left to right: Control, STZ.

Figure 4D

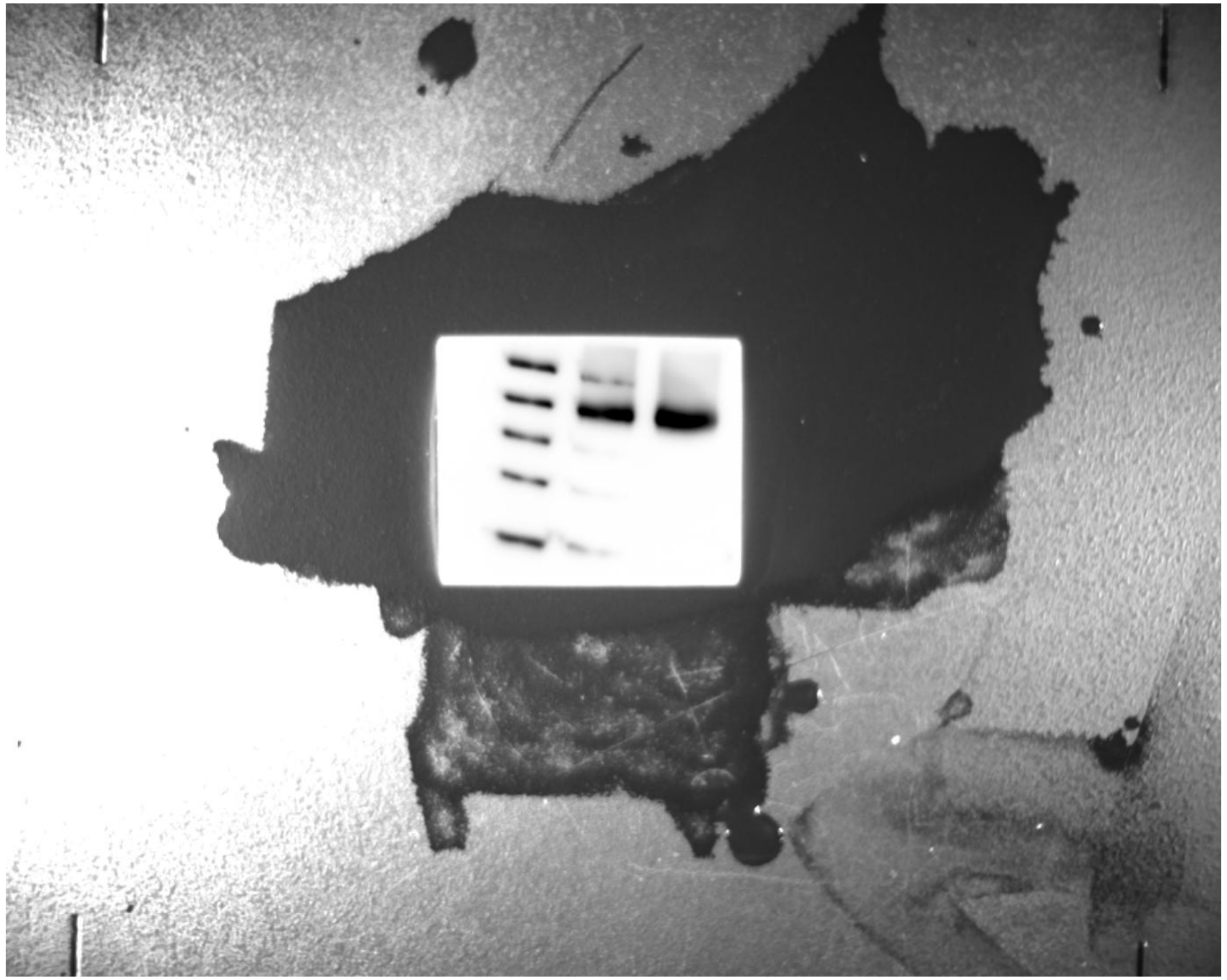

The original Western Blot images of IGF2BP2 in Figure 4D. From left to right: Control, STZ.

Figure 4D

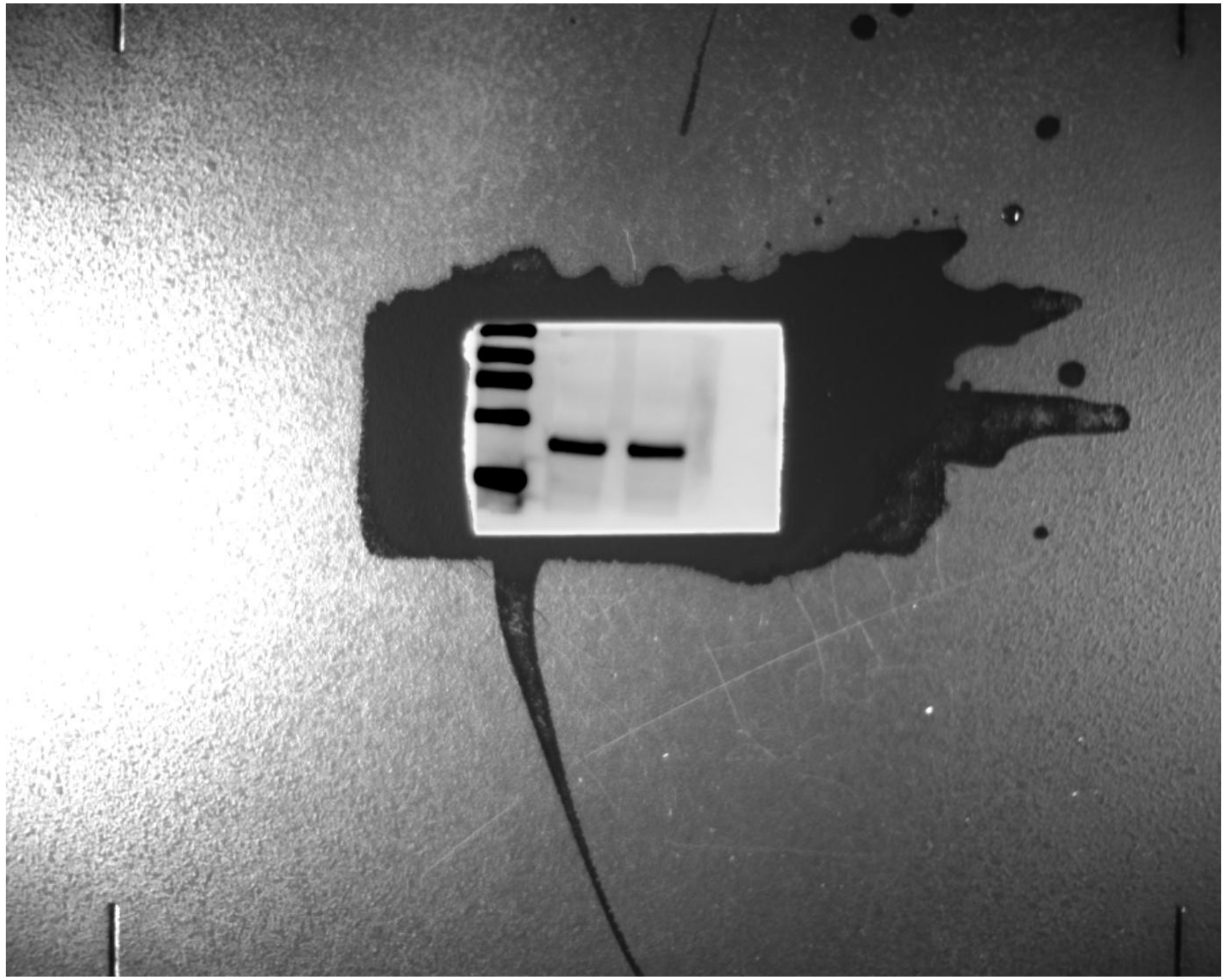

The original Western Blot images of GAPDH in Figure 4D. From left to right: Control, STZ.

Figure 8F

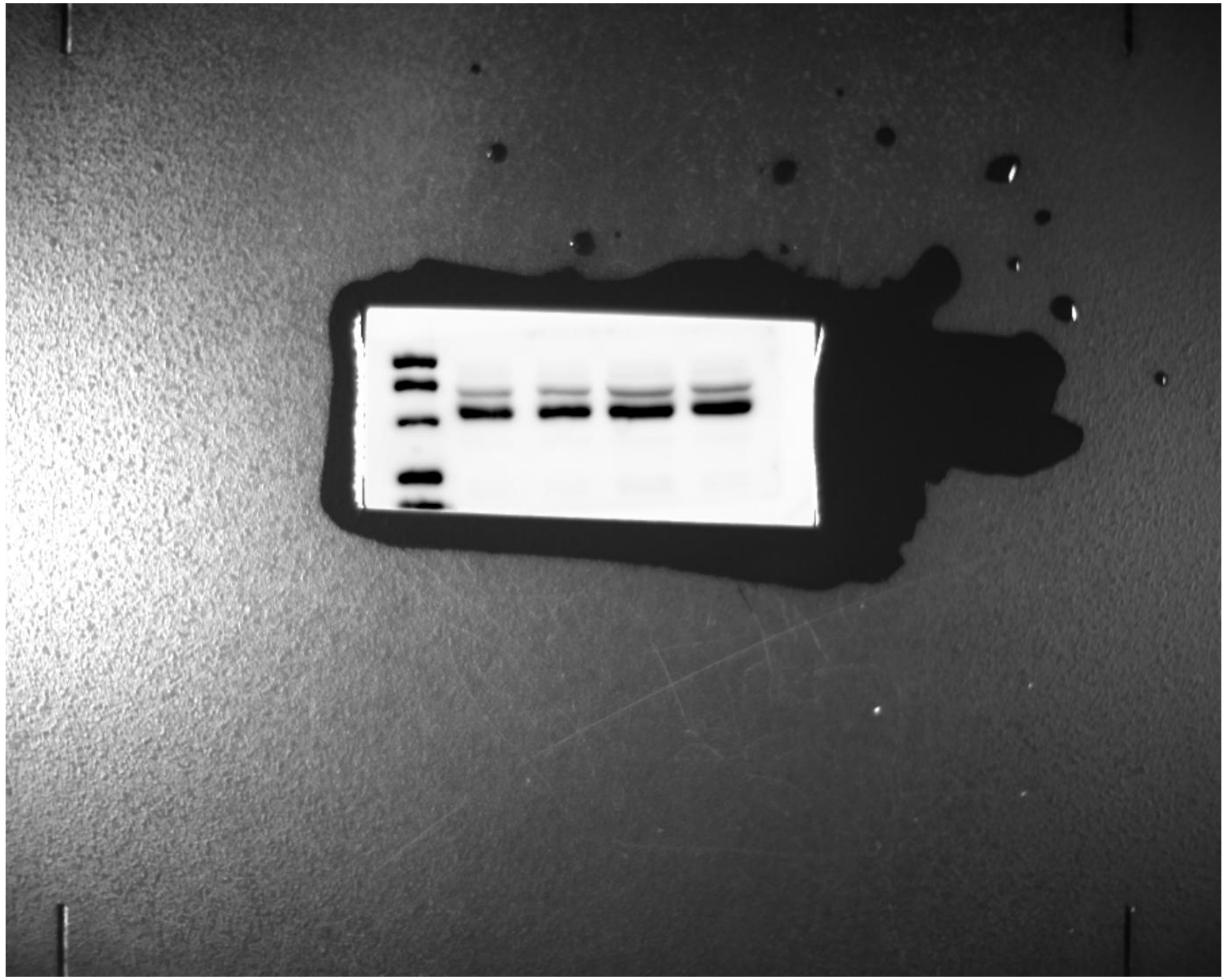

The original Western Blot images of TGF-β in Figure 8F. From left to right: Control, Cyn, STZ, STZ+Cyn.

Figure 8F

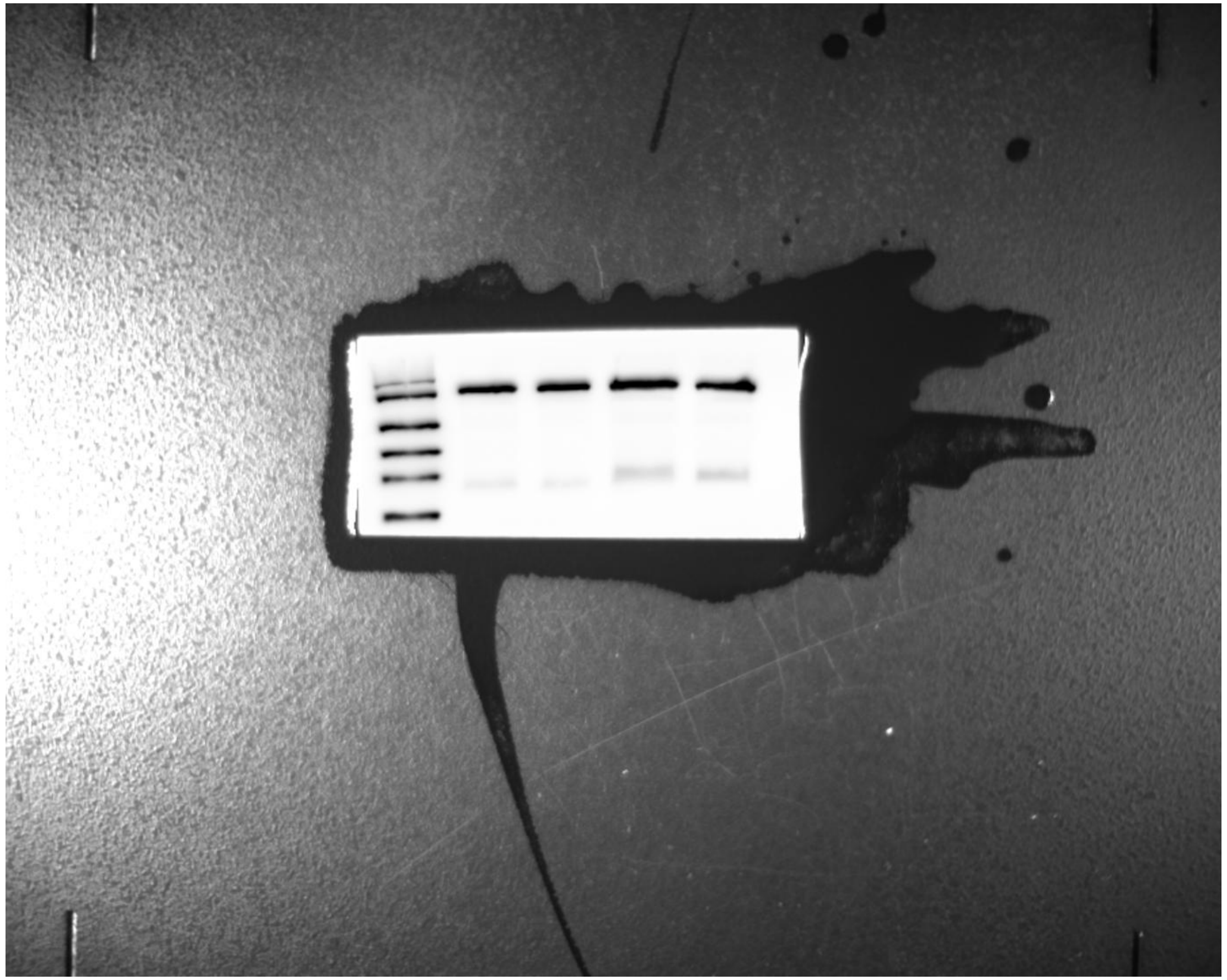

The original Western Blot images of COL-I in Figure 8F. From left to right: Control, Cyn, STZ, STZ+Cyn.

Figure 8F

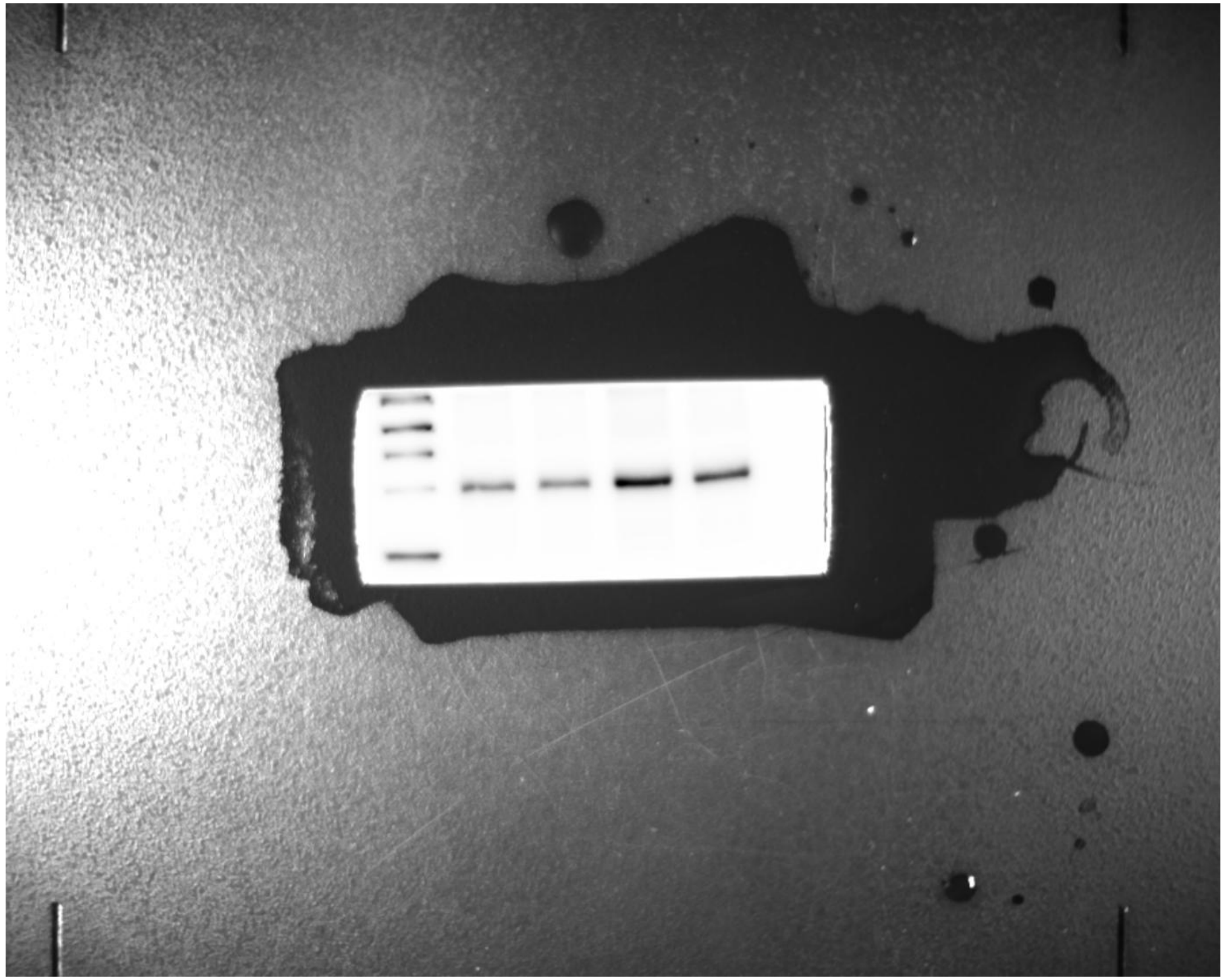

The original Western Blot images of α-SMA in Figure 8F. From left to right: Control, Cyn, STZ, STZ+Cyn.

Figure 8F

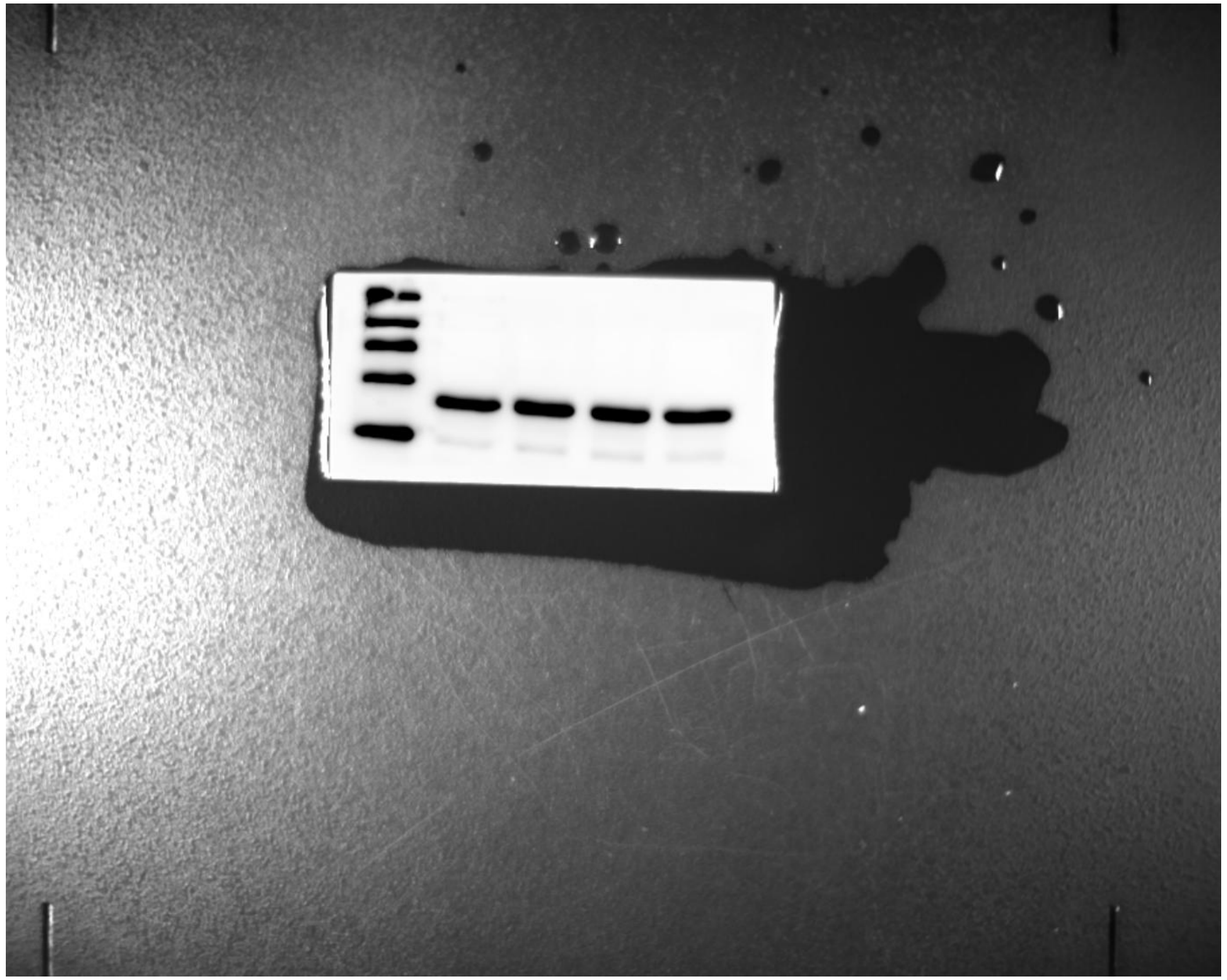

The original Western Blot images of GAPDH in Figure 8F. From left to right: Control, Cyn, STZ, STZ+Cyn.
