# Supplementary material for: Integrated transcriptomic and Mendelian randomization analysis identifies novel biomarkers for type 2 diabetes–associated cardiac dysfunction: cynaropicrin as a candidate intervention
Source: Naunyn Schmiedebergs Arch Pharmacol. 2026 Jul 10;399(13):20885–902. doi: 10.1007/s00210-026-05585-2 (PMC13695668; doi:10.1007/s00210-026-05585-2)
Supplement: Supplementary file 2 [file 210_2026_5585_MOESM2_ESM.docx]

| Gene | Primer | Sequence (5′–3′) |
| --- | --- | --- |
| PPIP5K2 | F | TTAAGAGCTGATGATGACGAGC |
|  | R | TTCTAGAGGACAGATGGATGG |
| IGF2BP2 | F | TCCCAAAGAAGAGGTGAAGCTG |
|  | R | CCTGCTGTACAATTTCCCTG |
| GAPDH | F | ACTCCCTCAAGATTGTCAGC |
|  | R | AGTTGCTGTTGAAGTCACAGG |

**Supplementary Table 1 Primer sequences used for quantitative real-time PCR**
